# Surface Modification of Mesoporous Silica Nanoparticles as a Means to Introduce Inherent Cancer‐Targeting Ability in a 3D Tumor Microenvironment

**DOI:** 10.1002/smsc.202400084

**Published:** 2024-07-08

**Authors:** Neeraj Prabhakar, Erica Långbacka, Ezgi Özliseli, Jesse Mattsson, Alaa Mahran, Ilida Suleymanova, Cecilia Sahlgren, Jessica M. Rosenholm, Malin Åkerfelt, Matthias Nees

**Affiliations:** ^1^ Pharmaceutical Sciences Laboratory, Faculty of Science and Engineering Åbo Akademi University Turku 20520 Finland; ^2^ Centre for Structural Systems Biology (CSSB) c/o DESY Notkestrasse 85 22607 Hamburg Germany; ^3^ Department of Physics University of Hamburg 20355 Hamburg Germany; ^4^ Biosciences Faculty of Science and Engineering Åbo Akademi University Turku 20520 Finland; ^5^ Institute of Biomedicine and FICAN West Cancer Centre University of Turku Turku 20520 Finland; ^6^ InFLAMES Research Flagship Center Åbo Akademi University Turku 20520 Finland; ^7^ Turku Bioscience Centre Åbo Akademi University and University of Turku Turku 20520 Finland; ^8^ Department of Pharmaceutics Faculty of Pharmacy Assiut University Assiut 71526 Egypt; ^9^ Faculty of Biological and Environmental Sciences Helsinki Institute of Life Science (HiLIFE) University of Helsinki Helsinki 00014 Finland; ^10^ Department of Biomedical Engineering Eindhoven University of Technology Eindhoven 5600 MB The Netherlands; ^11^ Institute for Complex Molecular Systems (ICMS) Eindhoven University of Technology Eindhoven 5600 MB The Netherlands; ^12^ Department of Biochemistry and Molecular Biology Medical University of Lublin Lublin 20‐093 Poland

**Keywords:** cancer‐associated fibroblast, extracellular matrix, mesoporous silica nanoparticles, organotypic 3D co‐culture, surface modification, targeted drug delivery, tumor microenvironment

## Abstract

Mesoporous silica nanoparticles (MSNs) have emerged as promising drug carriers that can facilitate targeted anticancer drug delivery, but efficiency studies relying on active targeting mechanisms remain elusive. This study implements in vitro 3D cocultures, so‐called microtissues, to model a physiologically relevant tumor microenvironment (TME) to examine the impact of surface‐modified MSNs without targeting ligands on the internalization, cargo delivery, and cargo release in tumor cells and cancer‐associated fibroblasts. Among these, acetylated MSNs most effectively localized in tumor cells in a 3D setting containing collagen, while other MSNs did so to a lesser degree, most likely due to remaining trapped in the extracellular matrix of the TME. Confocal imaging of hydrophobic model drug‐loaded MSNs demonstrated effective cargo release predominantly in tumor cells, both in 2D and 3D cocultures. MSN‐mediated delivery of an anticancer drug in the microtissues exhibited a significant reduction in tumor organoid size and enhanced the tumor‐specific cytotoxic effects of a γ‐secretase inhibitor, compared to the highly hydrophobic drug in free form. This inherent targeting potential suggests reduced off‐target effects and increased drug efficacy, showcasing the promise of surface modification of MSNs as a means of direct cell‐specific targeting and delivery for precise and successful targeted drug delivery.

## Graphical abstract

Mesoporous silica nanoparticles with a polyethylene imine surface coating (MSN‐PEI) do not penetrate the collagen matrix or enter cells in 3D. Meanwhile, acetylated MSN‐PEI (MSN‐PEI‐ACA) successfully moves through the matrix and specifically enters tumor cells, but not the surrounding tissue. The particles release their drug cargo inside the tumor cells, harming only these. This way, MSN‐PEI‐ACA particles constitute a drug carrier system for tumor‐specific, more effective anticancer therapy.

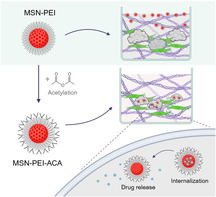

## 1 Introduction

Nanoparticles have shown significant potential in the medical field to yield improved cancer therapies by providing tissue‐ or cell‐type‐specific delivery that facilitates decreased cytotoxicity and side effects. Additionally, nanoparticles can overcome multidrug resistance by increasing bioavailability and lowering the required dosing for the therapeutic drug at the target site via targeted delivery and target‐specific (controlled or selective) drug release.^[^ ^1^, ^2^ ^]^ Among these, mesoporous silica nanoparticles (MSNs) have been under extensive research during the last two decades, especially for their targeted drug delivery potential, but MSNs are yet to be approved for clinical use.^[^ ^3^ ^]^ MSNs have a high specific surface area and pore volume along with tunable pore size, allowing them to carry high payloads of poorly water‐soluble drugs.^[^ ^4^, ^5^ ^]^ Nanoparticles can in general lower the required dosing by improving drug availability at the target site via targeted delivery and target‐specific, controlled drug release, thus presenting huge clinical value^[^ ^1^ ^]^. Here, the high modifiability of MSNs enables precise tailoring for specific applications and needs. MSNs consist of a porous silica core that can be functionalized with a surface coating or via direct conjugation of functional groups to the surface silanols, which can subsequently be further modified by the addition of active moieties in the quest for more cell‐specific targeting.^[^ ^3^, ^4^ ^]^

The surface coating and/or functionalization affect the particle size and net surface charge, which in turn alters the behavior of the MSN in a physiological setting. For example, non‐functionalized (native) MSNs have shown some toxicity on immune cells and the reticuloendothelial system.^[^ ^6^, ^7^ ^]^ This can be circumvented with the aid of proper surface functionalization. Hence, a plethora of surface modifications have been tested for better tailoring of MSNs for specific purposes. For instance, polyethylene glycol functionalized (PEGylated) MSNs exhibit a longer circulation time in vivo, lower liver accumulation and toxicity, as well as lower extent of protein binding (opsonization) compared to plain MSNs.^[^ ^8^, ^9^ ^]^ Polyethylene imine (PEI) functionalized MSNs show a higher cellular internalization compared to, e.g., PEGylated MSNs due to their positive net surface charge, enabling strong electrostatic interactions with the negatively charged cell membrane.^[^ ^10^, ^11^, ^12^, ^13^ ^]^ Nevertheless, too high of a positive surface charge density could translate into cytotoxicity, which is why plain PEI cannot be used as a delivery tool in vivo despite being the most effective gene transfection tool in vitro. Even though the mechanistic basis for the well‐known cytotoxicity of PEI is not fully understood, it is quite evidently at least partly connected to its exceptionally high positive charge density. It has both been shown that branched PEI can lead to higher cytotoxicity than linear PEI^[^ ^14^ ^]^ (with lower charge density due to its linear structure), as well as lower molecular weight PEI generally being less toxic than high molecular weight PEI.^[^ ^15^ ^]^ To further support this notion, it has been shown that derivatizing the primary amine groups of PEI with, e.g., PEG (simultaneously capping or at least reducing the positive charge) reduces its toxicity.^[^ ^16^ ^]^ The same effect may be the reason why the cytotoxic effects of PEI can be reduced when used as a component in nanoparticle designs.^[^ ^17^ ^]^ Nevertheless, too efficient cellular uptake can also lead to dose‐dependent toxicity effects, which still can be of benefit in terms of dose reduction.^[^ ^18^ ^]^ In any case, the common conjecture that nanoparticles with a net positive surface charge perform better than their negative counterparts in terms of cellular uptake has rendered them the focus of interest in many nanomedicine studies, despite the toxicity risks associated with a high positive surface charge.

The tumor microenvironment (TME) represents the immediate surroundings of the tumor in which tumor cells are embedded in solid cancers, and should be recapitulated in any representative and physiologically relevant tumor model. The TME significantly contributes to a vast array of oncogenic processes, such as tumor initiation and progression, local invasion and distant metastasis, as well as tumor dormancy and acquired chemoresistance.^[^ ^19^, ^20^, ^21^ ^]^ In addition to the tumor cells, the TME of epithelial cancer types, including breast and prostate cancers, also consists of other cancer‐promoting cells such as cancer‐associated fibroblasts (CAFs). CAFs come in multiple subtypes. They are resident fibroblasts that have been activated by tumor cells to facilitate tumor progression by producing matrix proteins such as collagens, and rearranging the structure and composition of the extracellular matrix (ECM).^[^ ^22^, ^23^, ^24^ ^]^ An excess of collagen fibers secreted by CAFs is the main cause of ECM stiffness and rigidity, and results in fibrotic or desmoplastic cancer tissues, which also hampers the penetration of anticancer drugs.^[^ ^25^, ^26^, ^27^ ^]^ Moreover, collagens have been shown to induce epithelial‐to‐mesenchymal transition (EMT),^[^ ^28^ ^]^ enhance angiogenesis,^[^ ^29^ ^]^ and increase tumor cell proliferation.^[^ ^27^, ^30^, ^31^ ^]^ Thus, it is vital to recapitulate the tissue architecture, cellular diversity and heterogeneity of the TME, as well as including ECM in the in vitro models used when screening small‐molecule compounds and nanoparticle delivery systems for potential cancer drug candidates that could assist in comprehending better cancer therapies.

The Notch signaling pathway is involved in cell–cell signaling within the TME. Notch receptors (NOTCH1‐4) and ligands are expressed by both cancer cells and CAFs.^[^ ^32^ ^]^ Aberration of this pathway in epithelial cancers has been linked to metastasis, increased aggressiveness, therapy resistance, and cell proliferation.^[^ ^33^, ^34^, ^35^, ^36^, ^37^, ^38^, ^39^ ^]^ Hence, Notch signaling is a desirable target for cancer chemotherapy in many cancer types, including castration‐resistant prostate cancer, and breast cancer.^[^ ^40^, ^41^ ^]^ The most frequently used class of Notch inhibitors are the γ‐secretase inhibitors (GSIs), which interfere with the last proteolytic step (S3) of Notch activation, which occurs after ligand binding.^[^ ^42^, ^43^ ^]^ Thus, the release of the transcriptionally active Notch intracellular domain (NICD) into the cytoplasm and nucleus is blocked. GSIs are typically hydrophobic molecules that have shown clinical potential (e.g., LY3039478, PF‐03 084 014, and AL101 in clinical trials as of 2021^[^ ^44^ ^]^). N‐[N‐(3,5‐difluorophenacetyl)‐l‐alanyl]‐S‐phenylglycine t‐butyl ester (DAPT) is a potent GSI that is extensively used to inhibit Notch signaling in vitro.^[^ ^43^, ^45^ ^]^ However, a critical downside of GSIs is their cause of severe side effects. Most notably, patients suffer from diarrhea and nausea following gastrointestinal toxicity as the result of inhibiting Notch functions in Notch‐dependent healthy tissues, and thus drug dosage and consequent therapeutic efficacy are limited.^[^ ^42^, ^46^ ^]^ GSIs in the treatment of cancer still remain an attractive therapeutic option; however, localized administration, lower dosages, or shorter treatment durations must be implemented to overcome these severe side effects.^[^ ^47^ ^]^

In this study, different MSN derivatives with distinct surface functionalities were tested in organotypic 3D co‐cultures containing tumor cells, CAFs, and collagen‐rich ECM. The 3D co‐cultures formed physiologically relevant tissue architectures, resulting in so‐called microtissues. We demonstrate that distinct surface modifications of MSNs exhibit specific affinity for tumor cells compared to fibroblasts, highlighting a selective targeting capability of MSNs in tissue‐like structures. Moreover, we show that localized delivery and efficacy of the GSI DAPT on the tumor was enhanced by surface acetylation of MSNs. These data underscore the potential of tumor‐specific targeting with MSNs for cancer therapy, and furthermore emphasize the possibility of implementing relevant models and screening approaches in nanomedicine research.

## 2 Results

### 2.1 MSN Surface Functionalization Resulted in Nontoxic Derivatives

**Figure 1:**
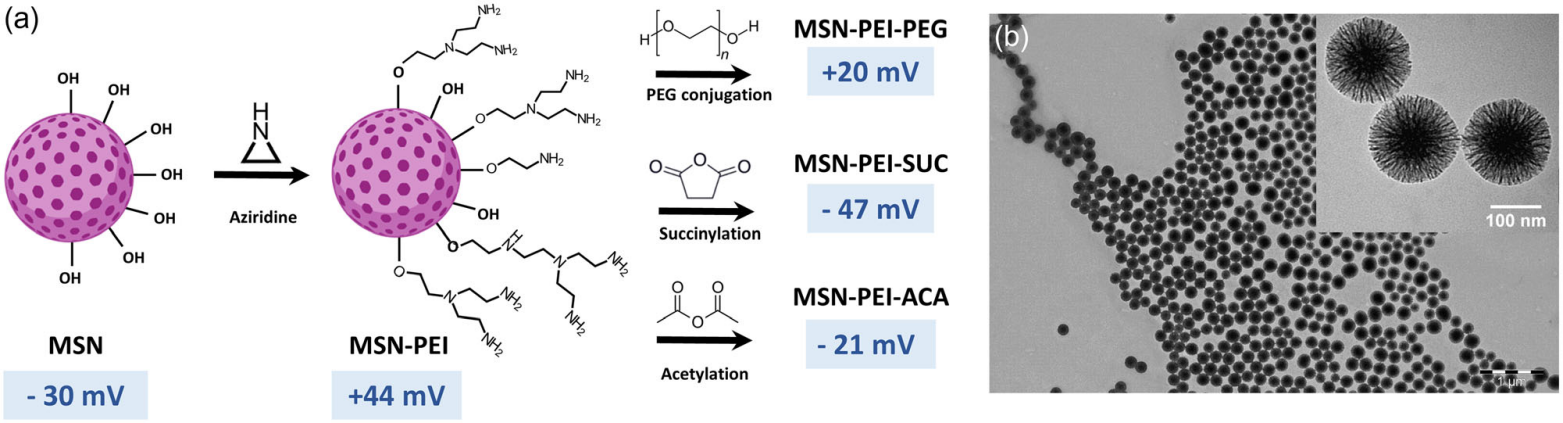
Physicochemical properties of core and surface‐modified MSNs. a) Schematic representation of the surface functionalization strategies employed for MSNs to yield a variety of surface modifications. b) TEM micrographs of MSNs showing an approximate average particle size of 100 nm with highly porous structure (inset).

To evaluate the effect of different MSN surface functionalizations in complex in vitro cell culture models, a variety of surface modifications were produced by functionalizing MSNs with different functional groups. This process of chemical conjugation via derivatization of PEI, which had been previously surface grown onto MSNs, is represented in **Figure** **1**a. Based on TEM images of core MSNs, the particles have a spherical shape and a diameter of around 100 nm, with a highly porous structure composed of radially aligned mesopores (Figure 1b). The TEM images furthermore revealed that these particles were not aggregated, i.e., both their dispersion and size distribution were homogeneous. Core MSNs with an initial ζ‐potential of −30 mV at neutral pH were surface‐grown with PEI to generate PEI‐MSNs with a ζ‐potential of +44 mV under the same conditions, i.e. the resultant ζ‐potential shift of ≈+75 mV clearly indicates that effective surface‐initiated polymerization of PEI had taken place. To further derivatize the PEI‐MSNs, PEGylation (+20 mV), acetylation (−21 mV), and succinylation (−47 mV) were undertaken. The two latter functionalization strategies derivatize the primary amine groups of PEI with succinic acid and acetyl groups via reaction with succinic and acetic anhydride. ζ‐potential measurements of the surface‐modified MSNs suspended in HEPES buffer at neutral pH validated the existence of successful, specific surface modifications on all defined MSN surfaces (**Table** **1**). To facilitate real‐time live‐cell microscopy detection in the subsequent cell‐based studies, all MSN derivatives were in situ labeled with the fluorophore tetramethylrhodamine‐isothiocyanate (TRITC) during synthesis.

**Table 1:** Physicochemical properties of core and surface‐modified MSNs.

MSN derivatives and their abbreviations	Hydrodynamic size (Z‐avg.) [nm]	PDI	ζ or Zeta‐potential [mV]
Core MSN (“MSN”)	206.8 ± 7	0.166	−29.6 ± 0.7
MSN‐PEI (“PEI”)	193.6 ± 4	0.193	43.6 ± 4
MSN‐PEI‐PEG (“PEG”)	204.1 ± 11.95	0.12	19.8 ± 8
MSN‐PEI‐ACA (“ACA”)	194.4 ± 4	0.156	−21.2 ± 0.95
MSN‐PEI‐SUC (“SUC”)	231.3 ± 9	0.226	−46.9 ± 9

DLS measurements were conducted in distilled water and electrokinetic measurements were conducted in 25 mM HEPES buffer, pH 7.2. MSN: mesoporous silica nanoparticle; PDI: polydispersity index; PEI: polyethyleneimine; PEG polyethylene glycol; ACA: acetyl; SUC: succinic acid.

Examination of hydrodynamic size (Z‐average), polydispersity index (PDI), and ζ‐potential values of surface‐functionalized MSNs in HEPES buffer solution (25 mM, pH 7.2) at the concentration of 0.1 mg mL^−1^ are detailed in Table 1. Toxicity evaluation of MSNs was conducted through viability analysis upon 24 h and 5‐day treatment on adenocarcinoma cells with 1, 10, and 50 μg mL^−1^ MSN derivatives. The earlier timepoint was included as a standard procedure, and the later one was included to match the length of treatment for MSN internalization studies. At 24 h, there was no significant toxicity of MSNs (in agreement with our previously published results^[^ ^48^, ^49^ ^]^) (Figure S1, Supporting Information). At 5 days, there was some toxicity at the 50 μg mL^−1^ concentration, most likely due to the long treatment time (Figure S2, Supporting Information).

### 2.2 Cellular Uptake of MSNs in 2D Co‐culture is Higher in Tumor Cells than in CAFs

**Figure 2:**
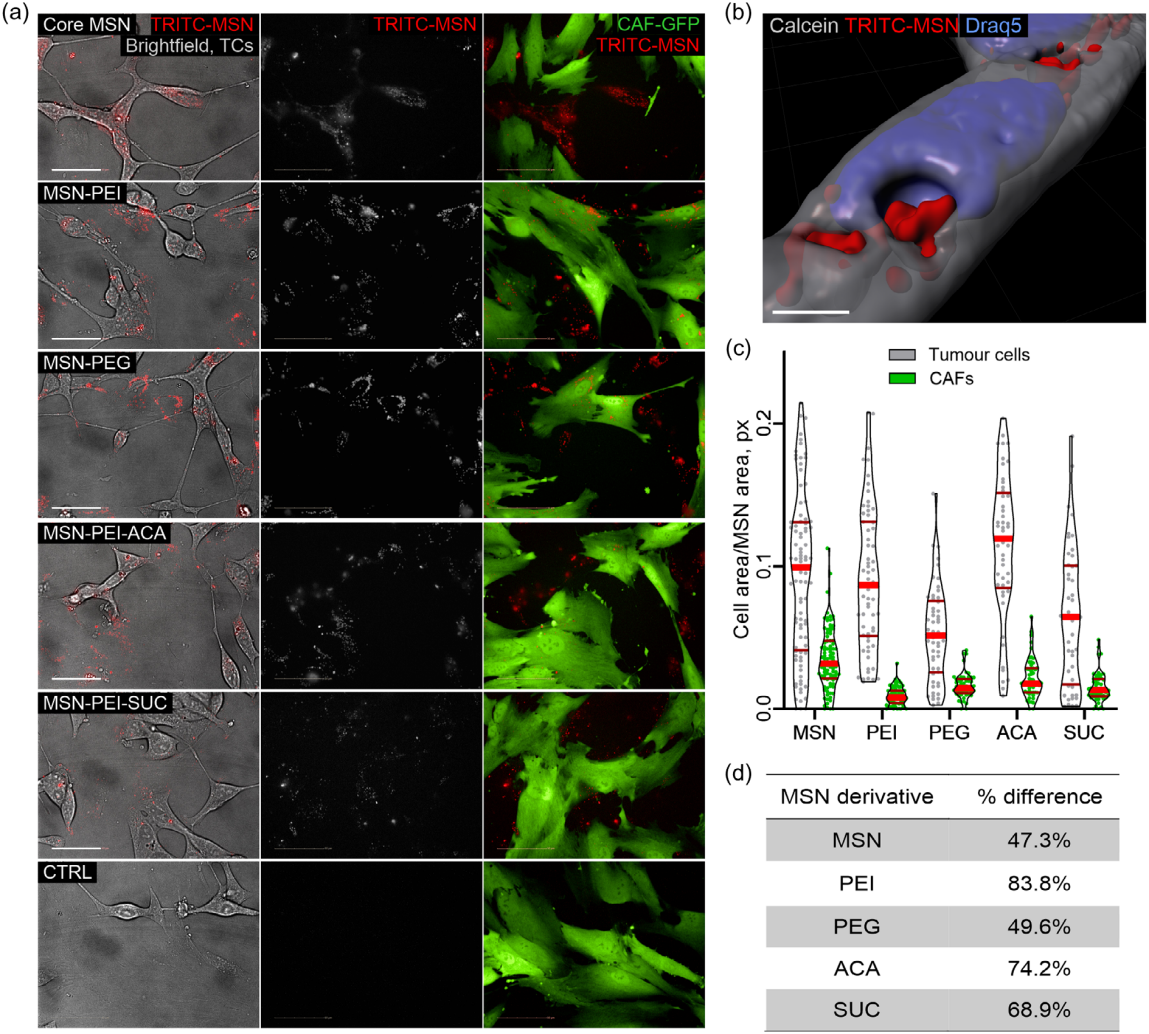
Cellular uptake of functionalized MSNs in 2D co‐cultures was more effective in tumor cells. Live‐cell confocal imaging showed that TRITC‐labeled nanoparticles (red) were effectively internalized into both LNCaP prostate cancer cells (transparent) and to a lower degree in GFP‐expressing CAFs (green). Image analysis revealed that all MSN derivatives were preferentially internalized by tumor cells. a) Confocal images are divided into brightfield (left panel) and 561 nm fluorescent channels to show the localization of MSNs in tumor cells. The 561 nm channel (middle panel) shows only MSNs, and the right panel (merged images of 561 and 488 nm channels) shows the localization of MSNs within CAFs. Linear ITF (i.e. brightness and contrast) was modified for optimal visualization, but not for image analysis. Scale bar = 50 μm. b) Surface rendering of a Z‐stack confocal image depicting MSN internalization into tumor cells. Calcein AM staining was captured at 488 nm (gray LUT), TRITC‐MSNs were captured at 561 nm (red LUT), and Draq5 nuclear staining was captured at 640 nm; scale bar = 10 μm. Surface rendering was created with Imaris software. c) Image analysis was performed for quantifying colocalization of MSNs inside tumor cells and CAFs. Quartiles are denoted by red lines. Statistical significance was analyzed with a student's *t*‐test with Bonferroni correction, *n* = 100 images/treatment, **** = *p* ≤ 0.0001. d) Difference in MSN localization observed between tumor cells and CAFs, expressed as percentage difference, derived by dividing the difference in uptake in tumor cells versus CAFs by total uptake and multiplying that ratio by 100%. Derivative names: MSN = Core MSN; PEI = MSN‐PEI; PEG = MSN‐PEI‐PEG; ACA = MSN‐PEI‐ACA; SUC = MSN‐PEI‐SUC.

To explore the internalization of surface‐functionalized MSNs, co‐cultures of the prostate cancer cell line LNCaP and prostate‐derived CAFs (immortalized cell line PF179T‐GFP with stable green fluorescence) were employed. 2D co‐cultures were treated with TRITC‐labeled MSNs (core MSNs, as well as PEI, PEG, ACA, and SUC surface modifications) at a concentration of 10 μg mL^−1^. Live‐cell confocal imaging performed 5 days after MSN addition showed that TRITC‐labeled nanoparticles were internalized by both LNCaP tumor cells and CAFs in 2D co‐cultures (**Figure** **2**a) and monocultures (Figure S3, Supporting Information, Supporting Video). This was also the case for breast cancer MCF7 cells, in 2D monoculture (Figure S4, Supporting Information) and co‐culture with PF179T‐GFP labeled CAFs (Figure S5, Supporting Information). The observed localization of MSNs was preferentially inside the cytoplasm (Figure 2b). The degree of particle internalization was measured by image analysis in 2D co‐cultures and was significantly higher in tumor cells compared to CAFs for all five MSN derivatives (Figure 2c). The most cell‐specific uptake was observed for MSN‐PEI with an 83% difference in favor of tumor cell internalization; however, the overall uptake was also the lowest for this derivative (Figure 2c,d) based on image analysis. The second‐highest difference was exhibited by MSN‐PEI‐ACA, which was internalized with a higher specificity toward tumor cells than toward CAFs (72% difference). This derivative also showed the overall highest degree of internalization (Figure 2c,d) and was therefore selected for subsequent studies in 3D organotypic cultures.

### 2.3 Only MSNs with a Net Negative Surface Charge Internalize into Tumor Organoids Cocultured with CAFs in an In Vitro TME

**Figure 3:**
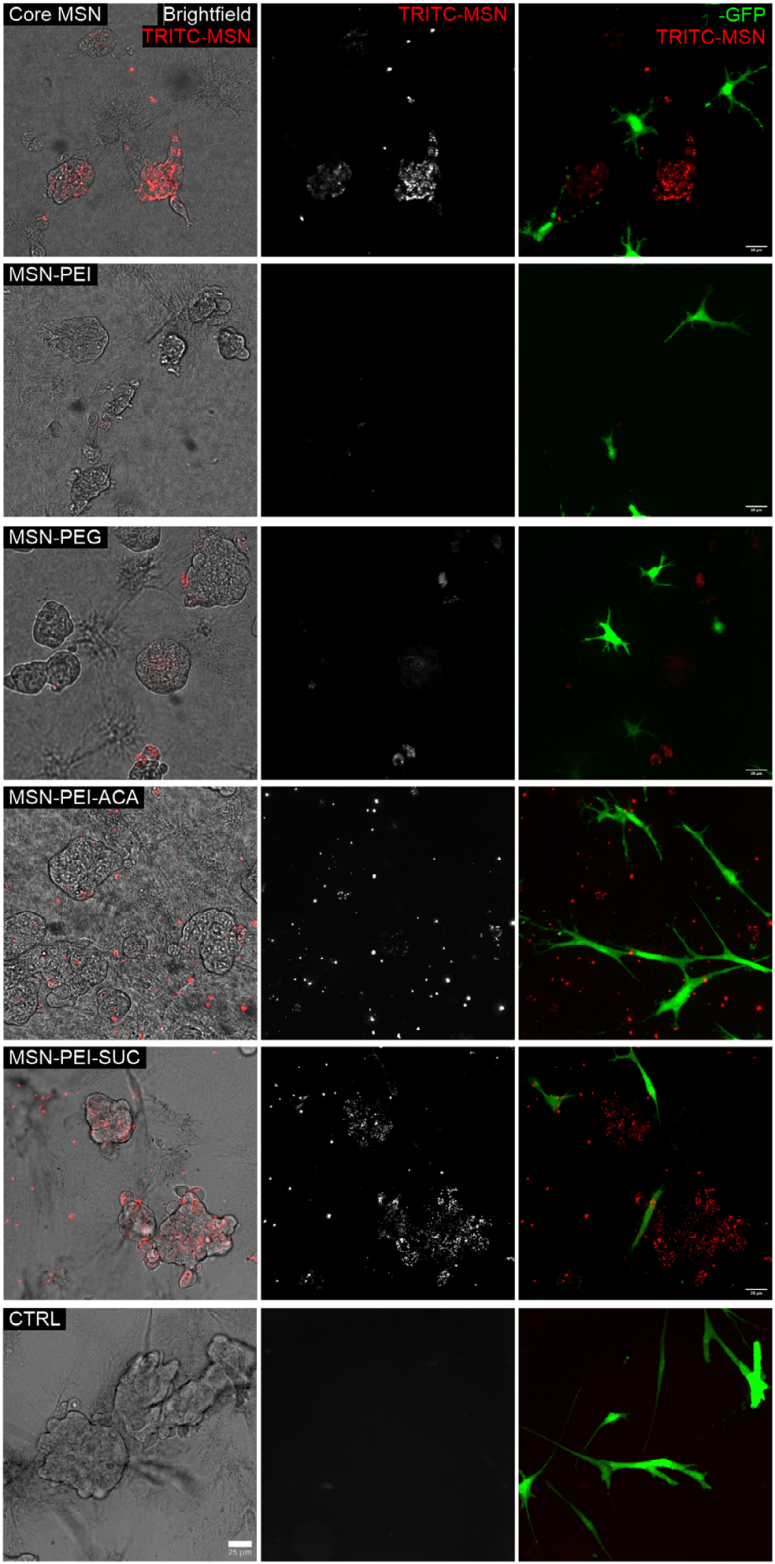
Plain MSNs, ACA, and SUC‐derivatized MSNs permeate collagen ECM and are preferentially internalized into tumor cells in 3D co‐cultures. Live‐cell confocal imaging showed that TRITC‐labeled MSNs (red) were differentially internalized into LNCaP prostate cancer organoids (transparent) and GFP‐expressing CAFs (green), depending on the surface modification. Confocal images captured on day 5 post‐treatment are split into brightfield and 561 nm channels to show the localization of MSNs within tumor cells. The 561 nm channel was used to visualize TRITC‐labeled MSNs, and the 561 nm combined with the 488 nm channel (right panel) shows the localization of MSNs in CAFs. Linear ITF was modified for optimal visualization. Control (CTRL) represents 3D cell cultures without MSN treatment. Scale bar = 25 μm.

Next, the surface‐modified MSN derivatives were tested to study whether surface modification affects cell‐specific uptake of MSNs in organotypic 3D co‐culture environments (LNCaP prostate tumor cells vs CAFs). We investigated the capability of MSNs to distinguish tumor cells from stromal cells using physiologically relevant collagen type I matrix in organotypic 3D co‐cultures that form microtissues. Live‐cell confocal microscopy was utilized to investigate the internalization of TRITC‐labeled MSN derivatives (core MSN, PEI, PEG, ACA, and SUC, all at 10 μg mL^−1^) by live cells. Our results showed that only MSNs with a net negative surface charge (core MSN, MSN‐PEI‐ACA, and SUC) permeated the collagen ECM and internalized into organoids, captured 5 days post‐treatment (**Figure** **3** and S6, Supporting Information). Significantly stronger fluorescent signals from these MSN derivatives could be detected inside tumor cells (transparent) compared with CAFs (green), indicating that MSNs with a net negative surface charge more successfully adhere to and penetrate cells in 3D. This result is also strengthened by matching observations in 3D monocultures at the same conditions (Figure S7, Supporting Information) and 3D co‐cultures of breast cancer cell line MCF7 with PF179T‐GFP CAFs (Figure S8, Supporting Information).

### 2.4 Model Drug (Dye) Release from MSN‐PEI‐ACA Nanoparticles Occurred Primarily in Tumor Cells in 2D and 3D Co‐cultures with CAFs

**Figure 4:**
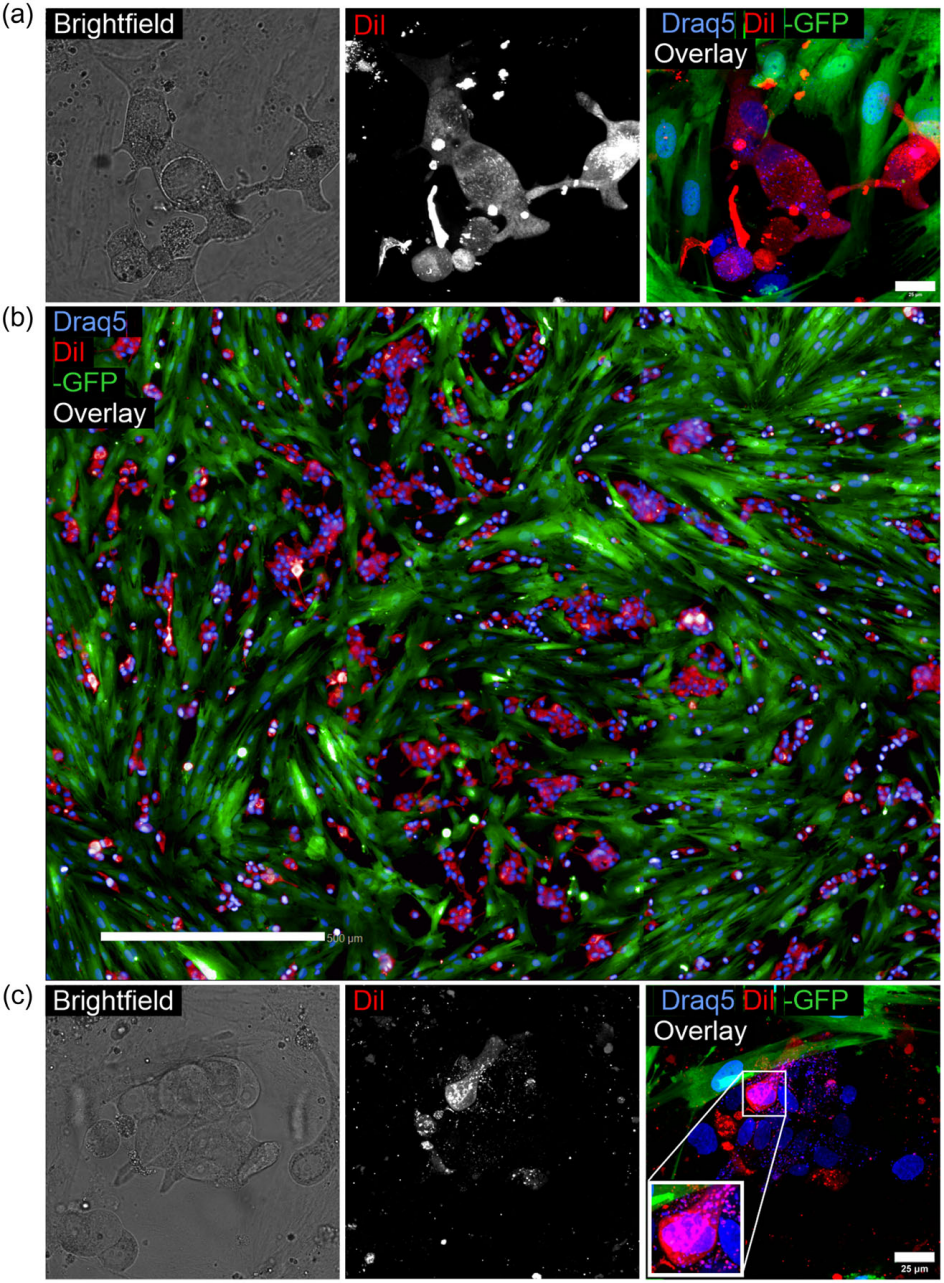
Intracellular delivery and release of a hydrophobic dye is more effective in tumor organoids compared to CAFs. MSN‐PEI‐ACA‐DiI dye release in 3D co‐cultures of LNCaP (transparent) and GFP‐expressing CAFs (green). Merged images consist of far‐red 640 nm channel (Draq5, blue LUT), 561 nm channel (DiI, red), and 488 nm channels (GFP, green). The ITF was modified for optimal visualization. Images were acquired on day 5 post‐MSN treatment. a) High‐magnification image of 2D co‐cultures, b) 2D co‐culture overview image composed by stitching together separate individual images. Imaging performed with Operetta confocal automatic imaging system, scale bar = 500 μm and c) 3D coculture image in collagen ECM generated by spinning disc confocal microscopy. Inset shows intracellular release of DiI cargo within a tumor cell, scale bar = 25 μm.

MSN‐PEI‐ACA particles were chosen for the delivery of hydrophobic cargo in the drug delivery experiments, based on their superior performance on internalization observed in 3D co‐cultures. We utilized MSN‐PEI‐ACA particles loaded with the hydrophobic fluorophore 1,1′‐dioctadecyl‐3,3,3′,3′‐tetramethylindocarbocyanine (DiI) (absorption 561 nm) as a model cargo for mimicking hydrophobic drug molecules while being able to follow the intracellular release real‐time.^[^ ^50^ ^]^ Thus, live‐cell confocal imaging was used to capture the presence and intracellular localization of DiI release. The resulting images showed that DiI is readily released from MSN‐PEI‐ACA particles inside cells co‐cultured in both 2D and 3D (**Figure** **4**). Moreover, dye release from MSNs seems to be strikingly specific to tumor cells as these unlabeled, transparent cells have become homogeneously red, while GFP‐CAFs remained green. Images of controls lacking treatment with MSN‐PEI‐ACA particles loaded with DiI indicated that the tumor cells remained transparent, suggesting the acquired fluorescence signal originates from the TRITC‐MSNs and is not affected by the autofluorescence from cells (Figure S8, Supporting Information). Similar results were observed for co‐cultures with MCF7 cells (Figure S10, Supporting Information). These results implicate that MSN‐PEI‐ACA not only penetrates the collagen‐based ECM and localizes primarily inside tumor cells, but is also able to release hydrophobic cargo specifically inside tumor cells and not CAFs. Thus, this surface‐modified MSN derivative represents a potential drug nanocarrier inherently capable of discerning between cell types, and specifically localizing with and releasing their hydrophobic cargo into tumor cells surrounded by dense ECM and stromal cells.

### 2.5 Cytotoxic Effects on Tumor Organoids versus CAFs Are More Pronounced by DAPT Released from MSN‐PEI‐ACA Than by DAPT in Free Form

**Figure 5:**
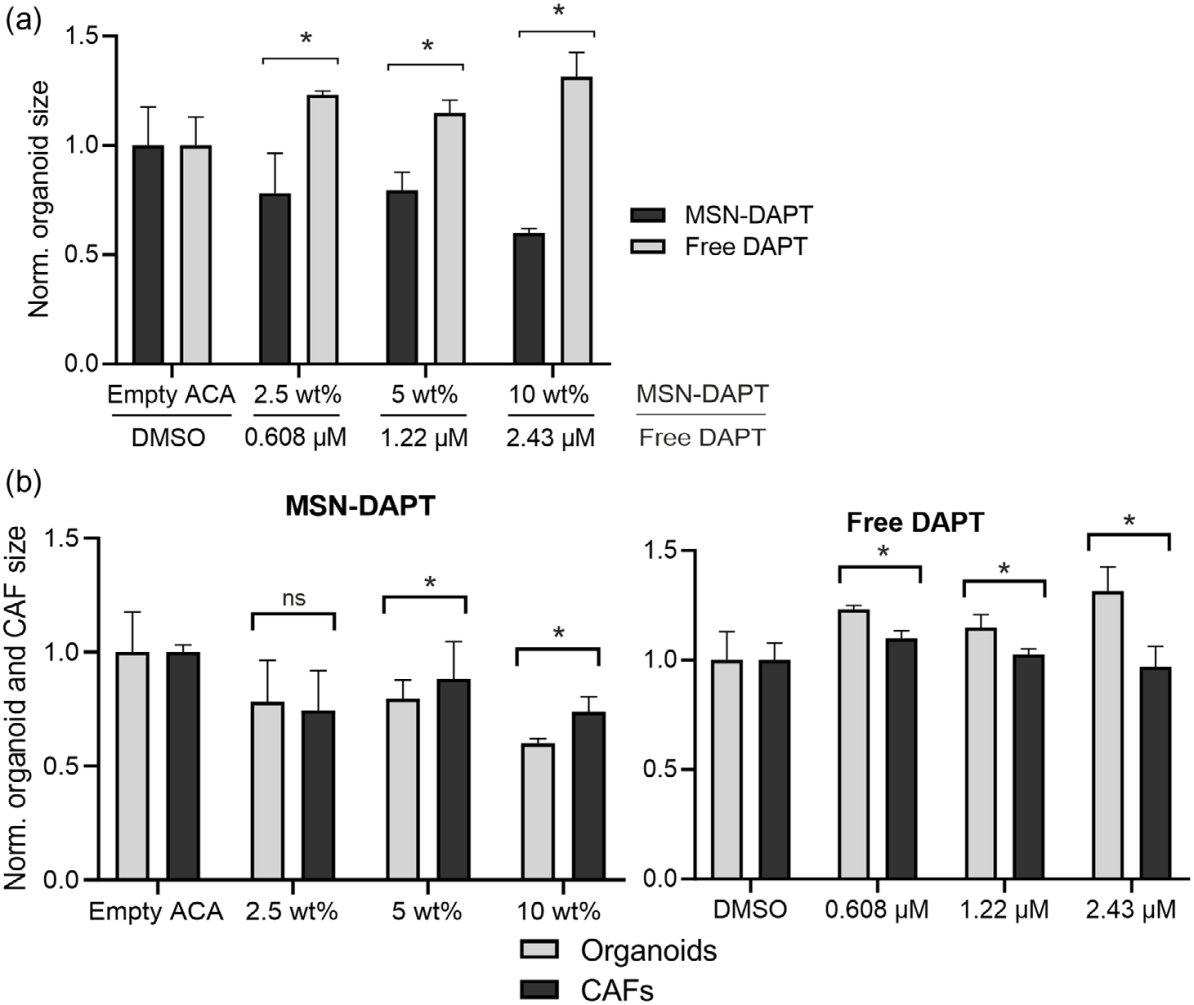
Inhibition of organoid growth by DAPT‐loaded MSN was more efficient than by free DAPT, specifically targeting tumor cells in a 3D co‐culture system. a) The size of LNCaP organoids in 3D co‐cultures shows significant differences in organoid growth and viability in response to DAPT treatment. DAPT delivered by MSN nanocarriers was more efficient in blocking organoid growth than DAPT in free form was. The y‐axis represents the size of organoids upon treatment, normalized to treatment with unloaded MSN‐PEI‐ACA or DMSO. b) Differential growth‐inhibitory effects of free DAPT versus MSN‐released DAPT are also visible when comparing organoid size with CAF size as a measure of cell proliferation. The *y*‐axis represents the size of organoids and CAFs upon treatment, normalized to treatment with unloaded MSN‐PEI‐ACA or DMSO. Two‐way ANOVA analysis with Bonferroni correction was performed, * = *p* ≤ 0.05, ns = nonsignificant. *n* = number of identified objects, around 150. Error bars indicate standard deviation. ACA = MSN‐PEI‐ACA.

To further confirm the selectivity of the MSN‐PEI‐ACA as a drug carrier in a biologically relevant 3D co‐culture model, LNCaP and CAFs co‐cultured in collagen ECM were treated with equivalent concentrations of DAPT in free form versus DAPT loaded in MSN‐PEI‐ACA nanoparticles. Drug release experiments were performed with unlabeled (transparent) MSNs loaded with 5 wt% (weight%) of DiI or DAPT at 2.5, 5, and 10 wt%, corresponding to 0.608, 1.216, and 2.432 μM DAPT, respectively (Figure S11, Supporting Information). The microtissues were fixed, stained by immunofluorescence, and imaged through confocal microscopy, followed by quantitative measurements of the size of LNCaP organoids compared to the size of CAFs by batch image analysis upon treatment. Treatment measurements were normalized to the respective vehicle controls (empty MSN‐PEI‐ACA particles devoid of drug, and 0.1% DMSO for free drug) to equalize possible nonspecific effects from the dispersing solution/vehicle. DAPT loading into MSN‐PEI‐ACA is shown in Figure S11, Supporting Information. DAPT‐loaded MSN‐PEI‐ACA significantly diminished the size of tumor organoids more effectively than DAPT in free form, and this effect correlated with increasing drug loads (10 wt% DAPT‐MSN‐ACA/2.43 μM free DAPT, **Figure** **5**a). Furthermore, DAPT delivery with MSN‐PEI‐ACA caused the size of the tumor organoids to decrease more than the size of CAFs (Figure 5b). Hence, MSN‐PEI‐ACA was evaluated to be able to overcome the limitations of free drugs and to cross cellular and extracellular barriers. In our experiments, DAPT‐loaded MSN was more effective than free DAPT in targeting tumor cells in complex organotypic 3D co‐cultures.

## 3 Discussion

Despite promising data from extensive nanoparticle research studies, many nanocarriers are failing to enter clinical trials, especially in oncology and epithelial cancers. Nanoparticles have widely been tested in simple 2D cell culture systems in vitro, and when transferred to in vivo systems, the results remain inconsistent due to the significant biological and systemic discrepancies between the two models. In this study, we implemented a physiologically relevant 3D model complemented with an ECM composed of collagen, which embedded tumor cells and CAFs, creating an in vitro TME. The results show that MSN drug‐carrying efficiency may be studied in systems closer to in vivo, circumventing actual animal models, and producing more translatable data from the get‐go. Moreover, we show that nanoparticle studies are compatible with high‐content, imaging‐based screening techniques, further improving experimental throughput and acquisition of valuable data. This also presents an opportunity for nanoparticles to enter clinical trials more quickly than in the past, as NIH has put forward a suggestion of no longer requiring in vivo testing during preclinical trials^[^ ^51^, ^52^ ^]^ and is actively promoting a shift toward the use of nonanimal models in general, which is in line with the 3R's principle (Replacement, Reduction, and Refinement of animal experimentation).^[^ ^53^ ^]^

MSN characteristics such as size, surface modification, and net surface charge have been extensively studied without reaching a consensus on the suitability of delivering anticancer agents in patients with epithelial cancers. This study involved toxicity and localization studies of native (nonfunctionalized) MSNs, as well as MSN‐PEI and its secondary functionalizations, i.e. succinylation, acetylation, and PEGylation of the surface‐grown PEI. In a 2D co‐culture setting, PEI‐functionalized and PEGylated MSNs showed the lowest overall efficiency of internalization by cells, while succinylated and acetylated MSN‐PEI showed the highest. In this setting, a striking result was the inherent tendency of all MSN derivatives to localize with significantly higher specificity to tumor cells rather than to CAFs. Targeting the tumor cells over the fibroblasts is also the main goal in cancer research, as tumor cells are the ones carrying the genetic mutations and can further progress, while CAFs are not genetically altered and do not form any metastases elsewhere. The MSN localization in tumor cells was observed in both 2D and 3D co‐cultures, and is in agreement with results from a study by Costa et al. published in 2013, conducted on 2D co‐cultures with MCF7 breast tumor cells and fibroblasts and chitosan‐histidine‐arginine‐polymer‐based nanoparticles.^[^ ^54^ ^]^ This is the only comparable study which, to our knowledge, has examined nanoparticle localization and uptake in a tumor/stroma co‐culture system, supported by a recent review aiming to outline the physiological factors that should be considered to bridge the translational gap between in vitro cell culture studies and in vivo studies in cancer nanomedicine.^[^ ^55^ ^]^

Our results demonstrate that the most successful MSN derivative was MSN‐PEI‐ACA, in terms of showing the highest degree of cellular uptake, the second highest difference in specific uptake into tumor cells over CAFs, and being able to internalize into cells cultured in collagen ECM. In this setting, PEI‐functionalized and PEGylated MSNs, which have generally been favored in studies based on 2D monolayer cell cultures and models,^[^ ^56^, ^57^ ^]^ were significantly less successful. This is an interesting observation given that in one of our previous studies, MSN‐PEI internalization into LNCaP organoids embedded in Matrigel ECM performed sufficiently well, suggesting that the derivatized counterparts studied here could potentially perform even better.^[^ ^58^ ^]^ Another previous study conducted in zebrafish revealed that aminosilane‐functionalized MSNs, MSN‐PEI, and its derivatives all displayed similar toxicity when directly microinjected into fish organs,^[^ ^18^ ^]^ whereas MSN‐PEI was considerably more toxic when added to the media surrounding the embryo. Namely, this course of action led to high fluorescent signals originating from the MSN‐PEI particles throughout the embryo, while the other particle types clearly aggregated on top, but did not penetrate the skin. This study thus clearly indicated that the passive penetrance through biological barriers is critical for toxicity evaluation in more complex settings than simple monolayer cell cultures. Thus, this strongly illustrates the high relevance and still unmet need for using more complex, physiologically and biologically relevant experimental models to evaluate the behavior of MSNs and other nanoparticles.

In addition, another article recently published by us further strengthens the indication of MSN‐PEI‐ACA being more promising over the earlier found successes of MSN‐PEI. Özliseli et al. (2023)^[^ ^59^ ^]^ tested the same set of MSN derivatives as this study in 2D and 3D collagen ECM, examining toxicity and internalization in myoblasts. Both in 2D and 3D at 6 h post‐treatment, MSN‐PEI‐ACA and MSN‐PEI‐SUC were the most internalized MSNs out of all the derivatives. Furthermore, we showed that ACA functionalization was superior to PEI‐ and PEGyation in terms of mobility in the collagen matrix, which should be one of the main reasons for its success also in the current study. Namely, while MSN‐PEI was immobilized in the matrix due to strong electrostatic interactions with the primarily negatively charged collagen, electrostatic repulsion exhibited by MSN‐PEI‐SUC or low interaction overall for MSN‐PEI‐ACA increased their mobility throughout the matrix, allowing cellular internalization. Contrary to common belief, PEGylation without any optimization does not automatically lead to enhanced colloidal stability or prevention of protein adsorption in a biological milieu. Thus, MSN‐PEI‐PEG formed aggregates and may also have become entangled in the polymeric (collagen) matrix. As a consequence, intracellular dye release was significantly more prevalent in ACA‐ and SUC‐treated cells after 48 h, as compared to PEI‐ and PEG‐treated cells. Finally, this study also showed the success of DAPT delivery to cells in 2D and 3D matrices. Given that PEI functionalized and PEGylated MSNs have previously shown success in in vivo studies,^[^ ^60^, ^61^, ^62^, ^63^, ^64^ ^]^ the current study not only highlights the importance of physiologically relevant testing platforms but also indicates interesting prospects for MSN‐PEI‐ACA as a delivery platform as well.

Our choice of model drug for loading into MSNs was mainly motivated by the need for DAPT to be administered to tumor cells by nanocarriers, as this hydrophobic compound is poorly water‐soluble and highly unstable in aqueous solutions. This strategy also represents a viable option to avoid extensive off‐target toxicity on healthy, Notch‐dependent tissues. Using DAPT as a drug model we have previously shown^[^ ^49^ ^]^ selective tumor‐cell specificity when treating mouse xenograft tumors in vivo, but in this case for larger PEI‐MSNs conjugated to folic acid (FA) as active targeting ligand. In this study, DAPT was carried to the tumor tissue by FA‐PEI‐MSNs, and induced stabilization of tumor size over 27 d. In contrast, xenograft tumors treated with an unloaded, drug‐free FA‐PEI‐MSNs tripled in size over the same time period. In comparison, treatment with free DAPT administered without a nanocarrier led to the tumor size doubling over 27 d, i.e., was considerably less effective than the nanoparticle formulation. Importantly, the same study also showed that nonspecific toxicity on the gastrointestinal tract of the mice was diminished when DAPT was carried by MSNs.^[^ ^49^ ^]^ The same particles were later evaluated in a chorioallantoic membrane xenograft assay^[^ ^61^ ^]^ whereby the results were found to correlate well with the previous in vivo study, again showing that more complex in vitro models potentially have higher predictive power, especially when addressing questions related to drug uptake and release in patient tissues (in vivo). In the current study, we found that in our 3D co‐culture model, DAPT is more efficiently and specifically acting on tumor cell proliferation and growth when carried by and released from “nontargeted” MSN‐PEI‐ACA nanoparticles, as compared to the same drug introduced in free form. This observation demonstrates an intrinsic capacity of surface‐modified MSNs to inherently and specifically target the tumor cells over nontumor components of the TME, thus potentially enabling higher and more specific treatment efficiency and circumventing commonly observed off‐target cytotoxicity in healthy cells and tissues. On this note, unloaded PEI‐MSNs have shown not only inherent targeting ability but also specific killing of glioblastoma stem cells,^[^ ^65^ ^]^ in this case, not only eliminating the need for an active targeting ligand but also a cytotoxic drug. Generally, it would be advantageous to utilize more complex multicellular and tissue‐like model systems to study the dynamics of MSNs in tissue‐like structures. The use of physiologically more relevant microtissues may also be a first step in the evaluation of the true potential of certain MSN preparations for targeted (chemo)therapy, and for the identification of the most promising nanocarriers among a range of possible modifications. Although animal experiments and also 2D monolayer cell culture settings still dominate cancer research, unsatisfactory research on clinical trials highlights the need for alternative models for nanotoxicity studies on a single‐cell level.

In conclusion, based on our results from 2D co‐cultures and TME including microtissues, we suggest that specifically designed MSNs can be introduced with intrinsic targeting ability in cancer nanomedicine also without the use of specific targeting ligands. Our MSN derivative MSN‐PEI‐ACA exhibited an inherent capacity of distinct targeting of the tumor cells over the stroma, alluding toward enhanced therapeutic efficiency and diminished off‐target toxicity. By using physiologically relevant TME‐like 3D models in evaluating nanoparticle performance, crucial design aspects can be revealed that contradict those derived solely from oversimplified 2D cell culture models, which severely hamper the translation of nanomedicines.

## 4 Experimental Section

4.1

4.1.1

##### MSN Synthesis and Characterization: Synthesis

MSNs were synthesized according to a previously published protocol, using a heterogeneous oil−water biphasic stratification system that yields a uniform nanoparticle size of ≈100 nm with radially aligned pores.^[^ ^66^ ^]^ Briefly, 24 mL cationic surfactant cetyltrimethylammonium chloride (CTAC, Sigma Aldrich, St. Louis, Missouri, United States) was mixed with 0.18 g basic catalyst triethylamine (TEA, Sigma Aldrich) in 36 mL water and the mixture was allowed to stir gently for 1 h at 60 °C. Next, 20 mL of 20 v/v% tetraethyl orthosilicate cyclohexane (TEOS‐cyclohexane, Sigma Aldrich) solution was carefully introduced to the solution as a silica source and the reaction was kept on gentle stirring for 18 h at 60 °C. The resulting MSNs were collected by centrifugation and template removal was achieved by an ion‐exchange method. The extraction solution was prepared by dissolving ammonium nitrate (Sigma Aldrich) in absolute ethanol to a final concentration of 0.6 wt%. MSNs were dispersed in the extraction solution by sonication and allowed to stir at 60 °C for 6 h after which they were collected by centrifugation. The extraction procedure was repeated twice to ensure efficient surfactant removal and cytocompatibility for biological studies. To prepare fluorescent MSNs, a solution of 1.5 mg mL^−1^ tetramethylrhodamine (TRITC, Sigma Aldrich) in ethanol was prepared and dissolved using sonication and allowed to conjugate with 0.02 mL (3‐aminopropyl) triethoxysilane (APTES, Sigma Aldrich) under vacuum for 2 h. Then, the dye‐silane conjugate solution was added following the introduction of 20% TEOS‐cyclohexane solution to the synthesis reaction as part of the silica source to yield inherently fluorescent MSNs.

MSN‐PEI was achieved by further functionalization of MSNs using aziridine to achieve a net positive surface charge as described in our previous studies.^[^ ^67^ ^]^ MSNs (100 mg) were dispersed in toluene (10 mL) and 5.2 μL acetic acid was introduced to the mixture as a catalyst. The reaction was initiated with the addition of 52 μL aziridine (Menadione, Spain) and allowed to proceed under stirring for 18 h at 35 °C. MSN‐PEI nanoparticles were collected by centrifugation and washed with ethanol twice. Furthermore, to investigate the effect of surface modification of nanoparticles, MSN‐PEI were derivatized with succinic anhydride (MSN‐PEI‐SUC), acetic anhydride (MSN‐PEI‐ACA), or mPEG (MSN‐PEI‐PEG).

MSN‐PEI‐SUC was prepared by dispersing the nanoparticles in absolute ethanol to the concentration of 2 mg mL^−1^ and introducing succinic anhydride to the solution with 100% excess by weight. The reaction was kept overnight at room temperature and then nanoparticles were collected by centrifugation and washed with absolute ethanol three times. MSN‐PEI‐ACA was synthesized through a similar method by introducing acetic anhydride with 100% excess by weight instead of succinic anhydride, and the same procedure was followed as explained above. Finally, MSN‐PEI‐PEG samples were obtained by introducing activated mPEG with 100 wt% excess to the reaction in chloroform and allowed to conjugate overnight. The mPEG activation procedure was carried out following a previously published protocol, wherein mPEG (5 kDa) was combined with catalytic amounts of hexamethylene diisocyanate (HMDI) in chloroform at high temperature to yield HMDI‐activated mPEG.^[^ ^68^ ^]^ The thus obtained MSN‐PEI‐PEG was then collected and washed with chloroform twice and resuspended in ethanol. All synthesized nanoparticles were stored in the fridge (+4 °C) as a suspension in absolute ethanol (10 mg mL^−1^) until further use.

##### Characterization of Functionalized MSNs

MSNs were characterized by measuring the hydrodynamic size, net surface charge (ζ‐potential), particle structure, and pore alignment to ensure reproducibility. Hydrodynamic size measurements were performed through dynamic light scattering (DLS) using Zetasizer NanoZS (Malvern Instrument Ltd., UK). MSNs were dispersed in deionized water to a concentration of 0.1 mg mL^−1^ using sonication, and all optical measurements were performed in disposable polystyrene cuvettes (Starstedt AG & Co., Germany). Zeta‐potential measurements were carried out using the same instrument by dispersing the nanoparticles in aqueous HEPES buffer (25 mM, pH 7.2) to ensure the equivalent pH between the samples, and were loaded into disposable folded capillary cells (DTS1070, Malvern, UK). DLS and ζ‐potential measurements were repeated three times and the average of these results was reported. Transmission electron microscopy (TEM) was utilized to validate the size, spherical morphology, and the formation of porous mesostructures. Ethanol‐dispersed nanoparticles were sonicated (at a concentration of 0.05 mg mL^−1^) and 10 μL of this suspension was deposited onto carbon‐coated copper grids (Ted Pella Inc., USA), which were allowed to air dry prior to imaging. Imaging was performed using a JEM‐1400 Plus Electron Microscope (JEOL, Japan) operating at 80 kV equipped with an OSIS Quemesa 11 Mpix bottom‐mounted digital camera.

##### Drug Loading into MSNs and Quantification

Drug loading into the pores of MSNs was performed by dispersing vacuum‐dried nanoparticles in the nonpolar solvent cyclohexane at a concentration of 2 mg mL^−1^ using a COVARIS S2‐focused ultrasonicator. Desired amounts of DAPT (Tocris Bioscience, Bristol, UK) (2.5 wt%, 5 wt%, and 10 wt%) or dialkylcarbocyanine membrane probe (DiI) as a hydrophobic model drug was directly added onto the suspension, whereafter the reaction mixture was ultrasonicated and agitated overnight at room temperature. The following day the MSNs were collected by centrifugation and vacuum dried overnight to eliminate residual solvent and stored at +4 °C until further use.

For quantification of drug loading, DAPT‐loaded nanoparticles were dispersed in methanol (0.5 mg mL^−1^) and sonicated for 2 h to ensure the leaching of loaded DAPT. The supernatant was collected by centrifugation and DAPT amount was measured using high‐performance liquid chromatography. The set‐up consisted of a UV detector (diode array) set at 222 nm, Inertsil ODS‐3 4.6 × 150 mm, and a 5 μm particle size column (GL Science). The flow rate was 1 mL min^−1^, and water and methanol were used as eluents. A linear gradient of 50% methanol to 100% methanol over 10 min was used for detection. The output signal was monitored and processed using Chemstation Software (designed by Agilent Technologies, Waldbronn, Germany) according to the calibration curve prepared with known concentrations. The calibration curve and the output signals of DAPT can be found in the supplementary information.

##### Cell Lines and 2D Culture Conditions

Human male prostate adenocarcinoma cell line LNCaP (CRL‐1740) and female breast carcinoma MCF7 (HTB‐22) cells were obtained from American Type Culture Collection (ATCC, Manassas, United States). The nontransformed, immortalized human prostate‐derived CAF line PF179T‐GFP was gifted by Professor Varda Rotter's group (Weizmann Institute, Rehovot, Israel). CAFs and MCF7 were propagated in GlutaMax DMEM (Thermo Fisher, Waltham, United States) supplemented with 10% fetal bovine serum (FBS, HyClone, Logan, United States), and 1% streptomycin/penicillin (HyClone). LNCaP cells were cultured in RPMI‐1640 (Thermo Fisher) supplemented with 10% FBS, 1% streptomycin/penicillin, and 1% L‐glutamine (Thermo Fisher). The cells were passaged for up to 40 passages and regularly tested for mycoplasma contamination.

##### Miniaturized Organotypic 3D Cocultures

3D organotypic cell cultures were performed as described previously.^[^ ^69^ ^]^ In short, cells were seeded between a bottom layer (3 mg mL^−1^) and an upper layer (1.5 mg mL^−1^) of bioactive ECM formed by collagen type I. Before use, collagen (BD Bioscience, Franklin Lakes, New Jersey, United States) was neutralized and buffered with base and buffering agents. LNCaP tumor cells were directly cocultured with PF179T‐GFP CAFs at 1000 and 800 cells/well, respectively, in a μ‐angiogenesis 96‐well plate (Ibidi, Fitchburg, Wisconsin, United States). This created highly reproducible organotypic cultures representing an in vitro TME. We refer to the multicellular structures formed from single cells embedded in the ECM as “organoids” to distinguish them from “spheroids,” which may form as the result of spontaneous reaggregation of larger numbers of cells in low attachment cultures.

##### Addition of MSNs and Compounds to 2D and 3D Cell Cultures

For evaluation of MSN toxicity, MSN derivatives were suspended in cell culture media to the final concentrations of 1, 10, and 50 μg mL^−1^. MSNs were sonicated for 15 min with intermittent vortexing before dilution and addition to cell cultures. The medium was changed after 72 h. For dye‐release evaluation (as hydrophobic drug delivery model), 10 μg mL^−1^ MSN diluted in cell media was added to cultures. For DAPT‐release evaluation, 10 μg mL^−1^ MSN‐PEI‐ACA with a theoretical loading of 2.5, 5, and 10 wt% DAPT was added to cell cultures. For free‐drug efficacy evaluation, free DAPT in DMSO was diluted to final concentrations of 0.608, 1.22, and 2.43 μM which is the effective concentration of the DAPT‐loaded MSN‐PEI‐ACA, and added to cell cultures. The selected DAPT concentrations are also within the active range that usually is used for Noch inhibition in vitro.^[^ ^59^, ^70^ ^]^ MSN and DAPT vehicles HEPES and DMSO, respectively, were added as controls at equivalent concentrations. For MSN‐DAPT experiments, empty, and unloaded MSNs were used as controls. 2D monocultures on uncoated plastic dishes were allowed to establish for one day between cell seeding and treatment addition; whereas this duration was 2 d for 2D cocultures with fibroblasts, and 3 d for 3D cultures.

##### Viability Measurements of 2D Monocultures: Confluency Measurements Using Live Cell Image Analysis

A high‐content real‐time imaging system IncuCyte S3 (Sartorius, Göttingen, Germany) and integrated software were used to measure confluency of 2D monocultures. 5000 LNCaP/well; 2000 CAFs/well; and 3000 MCF7/well were plated in 50 μL complete DMEM in 96 well plates. MSN treatments were added the following day in 50 μL DMEM, resulting in a final well volume of 100 μL. Brightfield imaging was performed on treatment day 5. Confluence values (in %) were normalized to controls and plotted for MSN toxicity evaluation.

##### Colorimetric Analysis of Cytotoxicity of 2D Monocultures

An additional way of measuring viability upon MSN treatment was utilizing a Cell Counting Kit‐8 (CCK‐8, Dojindo, Rockville, United States). Cultures were prepared as for confluency measurements. At the experiment endpoint (24 h and day 5), CCK‐8 reagent was added at 10% of the well content volume. The plates were allowed to incubate at 37 °C for 30–120 min, depending on the cell line, and the absorbance was measured for 0.1 s at 450 nm in a Victor 2.0 spectrophotometer (Perkin Elmer).

##### Confocal Imaging

Confocal images were acquired with a Marianas CSU‐W1 spinning disc (50 μm pinholes, Intelligent Image Innovations Inc., 3i, Denver, United States) equipped with a Photometrics Prime BSI sCMOS camera (Tucson, United States), SlideBook 6 (3i), and 5x Zeiss EC Plan‐Neofluar (0.16 NA), 40x Zeiss LD Plan‐Neofluar (0.6 NA), and 63x oil‐immersion Zeiss Plan‐Apochromat (1.4 NA) objectives. For high‐content imaging of 2D cultures, an Operetta CLS imaging system (Perkin Elmer, Waltham, United States) setup was used equipped with integrated 20x (0.75 NA) and 63x (0.9 NA) objectives, a pinhole spinning disc, a CCD camera, and Harmony 3.5.1 (Perkin Elmer) image acquisition and analysis software. In addition, a Leica TCS SP5 confocal microscope (Leica Microsystems, Wetzlar, Germany) integrated with long‐working distance objectives (HCX PL APO 63x/1.20 W CORR CS, Leica Microsystems, Wetzlar, Germany) was utilized. The MSNs labeled with TRITC were excited with a 561 nm diode laser and fluorescence was collected at 585–610 nm with photomultiplier tubes. The brightfield channel was used for visualizing 3D tumor organoids.

##### Live‐Cell Imaging and Analysis

Live‐cell confocal imaging was made possible by the use of GFP‐transformed CAFs and live‐cell‐compatible nuclear dye Draq5 (far‐red, 640 nm, Biotium, Fremont, California, US). Imaging transparent, unlabeled tumor cells was performed with brightfield settings, while fluorophore‐based microscopy was conducted with lasers at 488, 561, and 640 nm. Live cell imaging was performed at 37 °C with a 5% CO_2_ atmosphere to maintain normal cell growth. Evaluation of MSN uptake in 2D was performed with the Operetta CLS imaging system set to capture 50–150 images per condition on days 3 and 5 after MSN addition to cultures. Live‐cell imaging of dye‐release was performed with a Marianas spinning disc confocal microscope. To perform a detailed, topical analysis of MSN localization in 3D cocultures and live cells, i.e., detecting objects of interest, a customized program was written using image processing libraries in MATLAB (Natick, Massachusetts, US). The program consisted of the following steps: first, for all images, intensity values were adjusted with the *imadjust* function; second, morphological functions were used to separate objects from each other with *impopen*; third, a *fill hole* function was used. When all operations were implemented, the final step was to measure the area of interest of the objects in pixels. All data points were used for analysis, while a few clear outliers that distorted the y‐axis scale were removed for data illustration.

##### Image Rendering in Imaris

3D rendering of images captured from 2D cultures was conducted in Imaris software (Oxford Instruments, Abingdon, UK). Z‐stack images were preprocessed in ImageJ (FIJI^[^ ^71^ ^]^) to optimize the intensity‐transfer function (ITF), and then opened in Imaris. Surface renderings for the red and blue channels (red: MSNs/TRITC, blue: nuclei/Draq5) were created, and the distance between each z‐slice was enlarged by a factor of five to improve 3D viewing. The green channel (Calcein‐AM life cell stain, Thermo Fisher) depicting the cytoplasm was assigned a gray look‐up table (LUT). Then, snapshots of the rendering were captured to show the precise localization of MSNs.

##### Immunofluorescence Staining of 3D Cocultures and Consequent Image Analysis

Organotypic 3D cocultures were fixed and permeabilized in the original 96‐well plate with 4% paraformaldehyde (Sigma Aldrich, St. Louis, United States) and 0.5% Triton X‐100 (Thermo Fisher). Subsequently, the reagents were washed with 1x PBS in three consecutive rounds. Primary antibodies (α‐pancytokeratin, mouse multiclonal, clone AE1/AE3 + 5D3, Abcam, ab86734; α‐vimentin, rabbit monoclonal, clone SP20, Abcam, ab16700) diluted in 3% BSA‐PBS (1:200) were incubated overnight. Primary antibodies were washed off with PBS, and secondary antibodies (Alexa Fluor647, α‐mouse, Thermo Fisher, A31571; Alexa Fluor 488, α‐rabbit, Thermo Fisher, A‐11 008 both diluted 1:200) and the nuclear dye Draq5 diluted 1:500 in 3% BSA‐PBS were allowed to incubate for 1 h. This suspension was washed off before imaging.

To quantitate data of MSN and DAPT treatments, image analysis was conducted on IF‐stained confocal images in FIJI.^[^ ^71^ ^]^ Prior to analysis, all images were normalized to a 5–95% range of intensity values and turned into maximum‐intensity projections in SlideBook 6 (3i). The channels (red, green, and blue LUTs) were split, and the tumor cell (pan‐cytokeratin, red LUT) channel was further processed by auto‐thresholding to separate the foreground from the background. The “*fill holes*”‐function was used to fill in the center of structures excluded from the threshold, and the “*watershedding*” algorithm was employed to separate adjacent touching structures. Next, the “*analyse particles*” function was used with a minimum size filter of 4 pixels. The fibroblast channel (vimentin, green LUT) was processed likewise by automated thresholding and the “*analyse particles*”‐function alone. The results of this analysis included the percent area of the organoids and fibroblasts/CAFs. To elucidate the number of structures, the same protocol was used, but the last step used the “*3D objects counter*”‐function with a size filter of a minimum of 20 pixels to remove any dead single cells, debris, and background noise. This function resulted in robust, reproducible data such as the number of objects, which was used as *n*. The blue channel of nuclei was not used for analysis. The quantitative data were plotted and statistically analyzed in Prism 8 (GraphPad Software Inc., San Diego, California, USA).

##### Statistical Analysis

Student's *t*‐test and two‐way ANOVA with Bonferroni correction were used for comparison of two or more experimental samples, respectively. Samples were normalized to their comparative counterparts. Sample sizes were calculated as a number of images or the number of structures in a screen, as denoted in the figure texts. Standard deviation was used for statistical analysis and error bars. Statistical significance is denoted by stars accordingly: ns = *p* > 0.05, * = *p* ≤ 0.05, ** = *p* ≤ 0.01, *** = *p* ≤ 0.001, **** = *p* ≤ 0.0001. Analysis of the data was performed utilizing GraphPad Prism 9 (GraphPad Software Inc.).

## Conflict of Interest

The authors declare no conflict of interest.

## Supporting information

Supplementary Material

## Data Availability

The data that support the findings of this study are available in the supplementary material of this article.
