# Supplementary material for: Surface Modification of Mesoporous Silica Nanoparticles as a Means to Introduce Inherent Cancer‐Targeting Ability in a 3D Tumor Microenvironment
Source: Small Sci. 2024 Jul 8;4(9):2400084. doi: 10.1002/smsc.202400084 (PMC11935100; doi:10.1002/smsc.202400084)
Supplement: Supplementary file 1 — Supplementary Material [file SMSC-4-2400084-s001.pdf]

Supplementary information:

Surface modification of mesoporous silica nanoparticles as a means to introduce inherent cancer targeting ability in a 3D tumor microenvironment

Neeraj Prabhakar[a,b,c#], Erica Långbacka[d,e,f,g#], Ezgi Özliseli[a], Jesse Mattsson[e], Alaa Mahran[a,h], Ilida Suleymanova[i], Cecilia Sahlgren[d,f,g,j,k], Jessica M. Rosenholm[a*], Malin Åkerfelt[d,e*], Matthias Nees[e,l*]

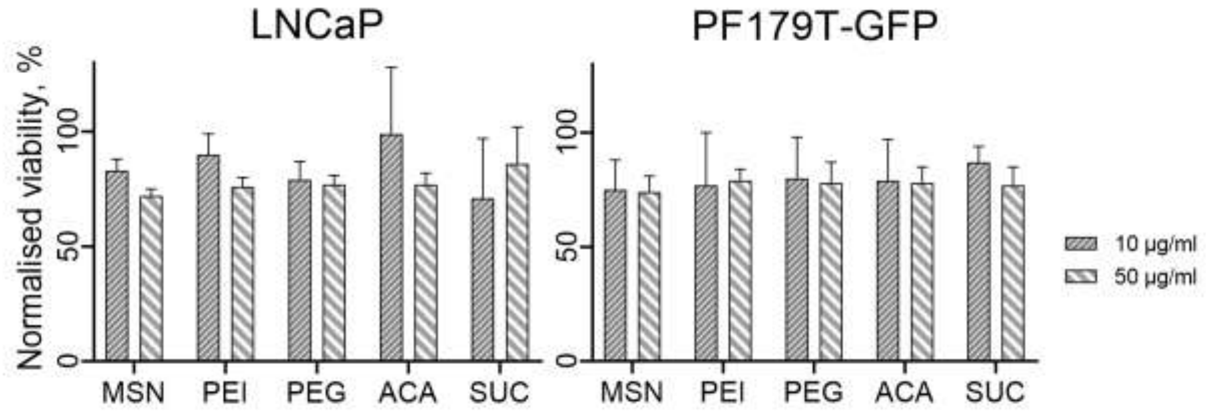

Supplementary Figure 1. Toxicity of MSNs remained low at a 10 and 50 µg/ml treatment for 24h, and no statistically significant differences between controls and treatments were found. 2D monocultures of LNCaP and PF179T-GFP were seeded at 5 000 cells/well into a 96 well plate and the following day treated with 10 and 50 µg/ml MSN derivatives. After 24h of incubation, the viability of cells was tested with a CCK-8 (WST8-based) colorimetric assay. The treatments were normalized to the untreated control (100%, not shown), and a two-way ANOVA was conducted comparing treatments to the control, of which all were non-significant, $p > 0.05$. $n = 3$ wells/treatment. Derivative names: MSN = Core MSN; PEI = MSN-PEI; PEG = MSN-PEI-PEG; ACA = MSN-PEI-ACA; SUC = MSN-PEI-SUC.

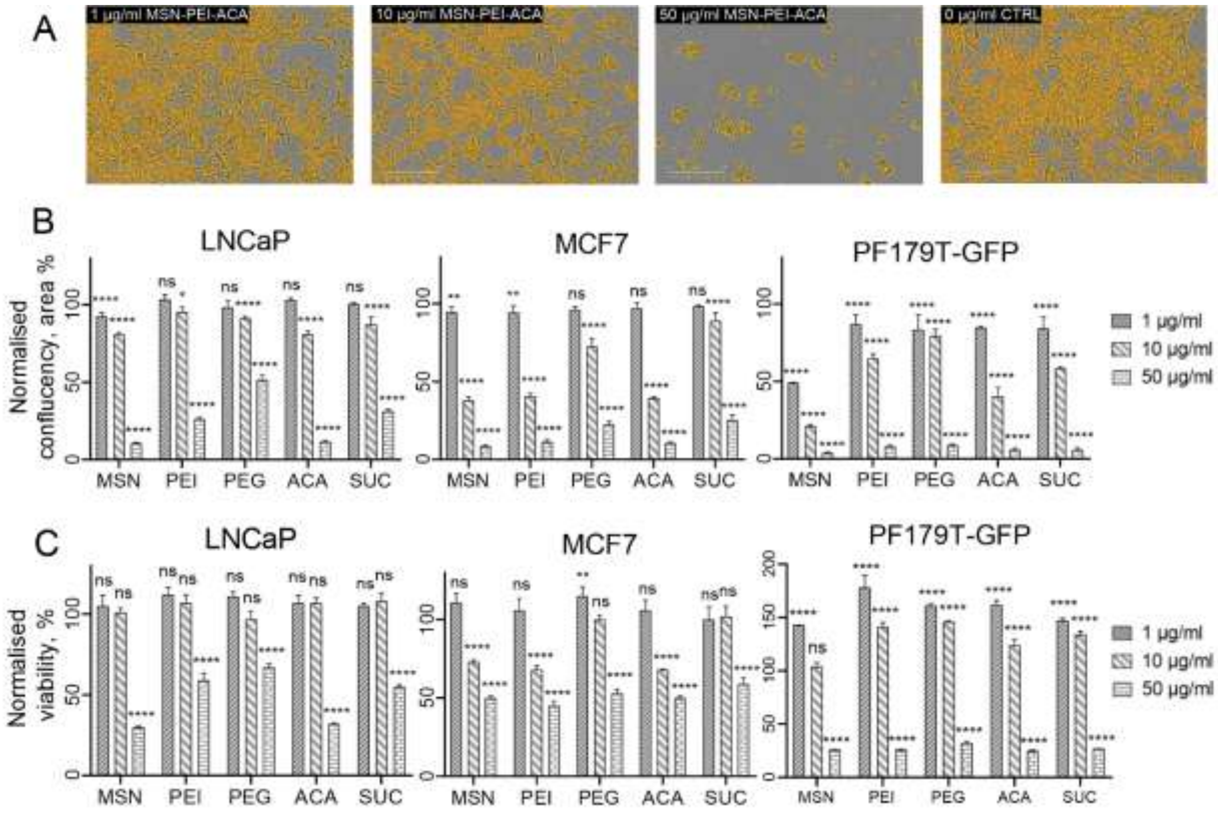

Supplementary Figure 2. Cells treated with MSN derivatives for 5 days showed dose-dependent toxicity. LNCaP, MCF7, and PF179T-GFP treated with 1, 10, and 50 µg/ml MSNs for 5 days exhibited the highest toxicity at 50 µg/ml, according to CCK-8 assay and confluency analysis. **A, B)** Confluence analysis conducted in integrated IncuCyte S3 software (Sartorius) was used to evaluate the cell viability upon MSN treatment. The yellow color in **A** represents measurement mask, representative images of LNCaP cells. 10 µg/ml MSN derivatives showed an acceptable effect on the adenocarcinoma cell toxicity. Conversely, 50 µg/ml was relatively toxic, based on the reduction in cell size upon apoptosis. **C)** Cell viability measured through CCK-8 at 450 nm (WST8-based) assay. The treatments were normalized to the untreated control (100%, not shown), and a two-way ANOVA was conducted comparing treatments to the control, n = 3 wells/treatment. Ns = $p > 0.05$, * = $p \leq 0.05$, ** = $p \leq 0.01$, *** = $p \leq 0.001$, **** = $p \leq 0.0001$. Derivative names: MSN = Core MSN; PEI = MSN-PEI; PEG = MSN-PEI-PEG; ACA = MSN-PEI-ACA; SUC = MSN-PEI-SUC.

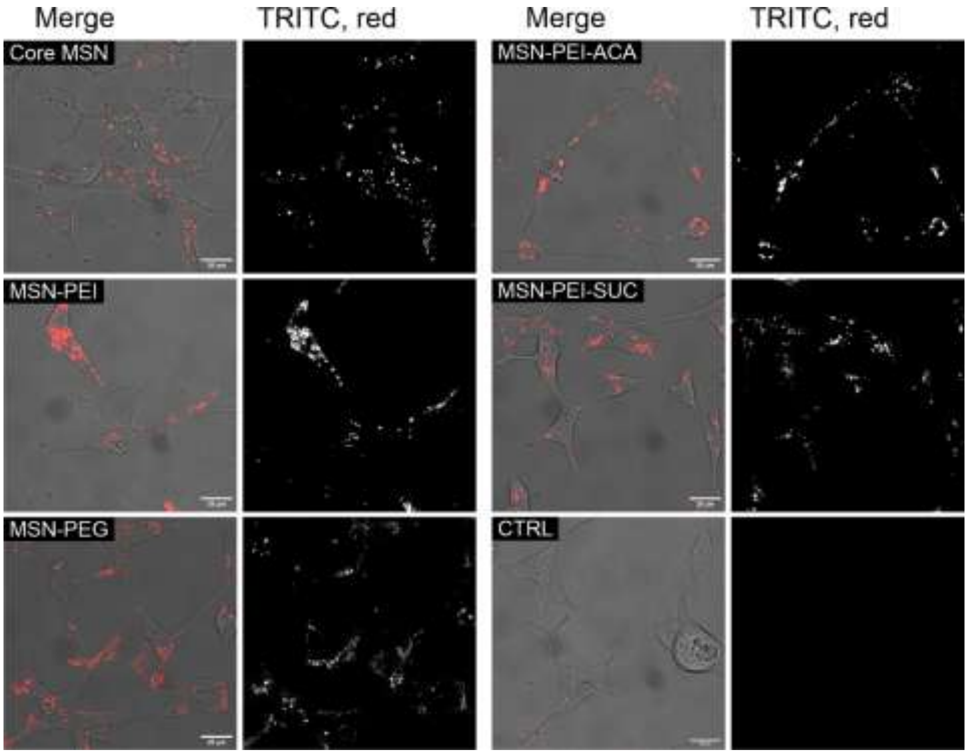

Supplementary Figure 3. Cellular uptake of MSN derivatives in 2D-monocultures containing LNCaP tumor cells. Confocal microscopy performed with Perkin Elmer Operetta CLS system, 60x. Linear ITF modified in integrated software (Harmony, Perkin Elmer) for visualization. Scale bar = 25 µm.

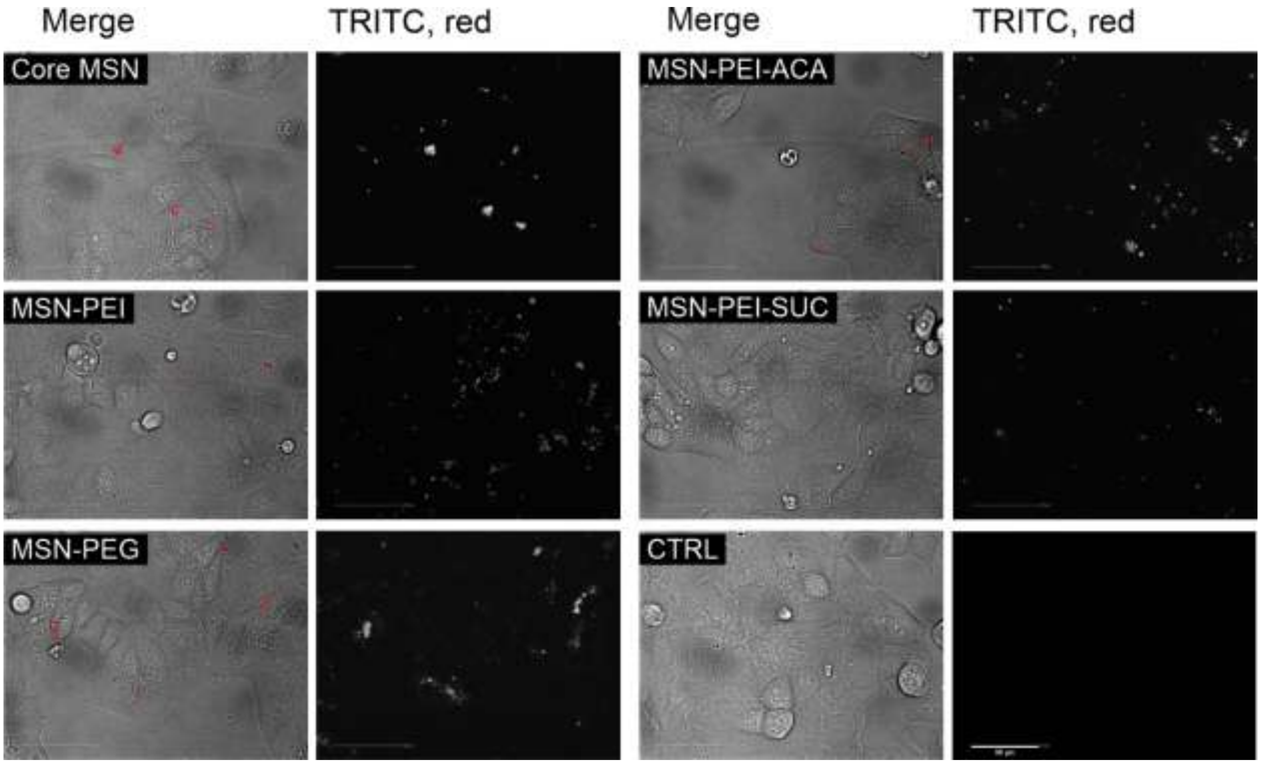

Supplementary Figure 4. MCF-7 cells in a 2D monoculture. Confocal microscopy performed with Perkin Elmer Operetta CLS system, 60x. Linear ITF modified in integrated software (Harmony, Perkin Elmer) for visualization. Scale bar = 50 µm.

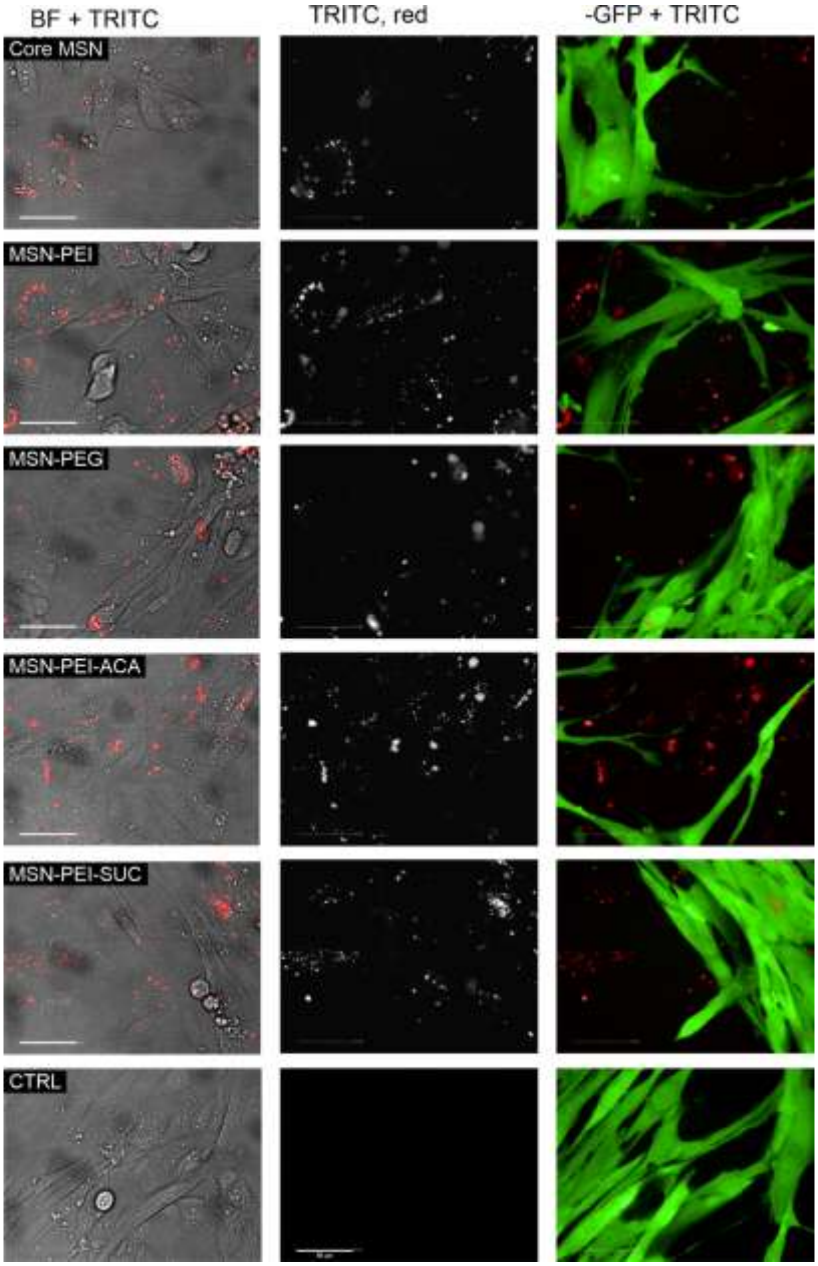

Supplementary Figure 5. MCF-7 cells (transparent) and CAFs (green) in a 2D co-culture treated with 10 µg/ml MSN derivatives. Live-cell confocal microscopy performed with Perkin Elmer Operetta CLS system, 60x. Linear ITF modified in integrated software (Harmony, Perkin Elmer) for visualization. Scale bar = 50 µm.

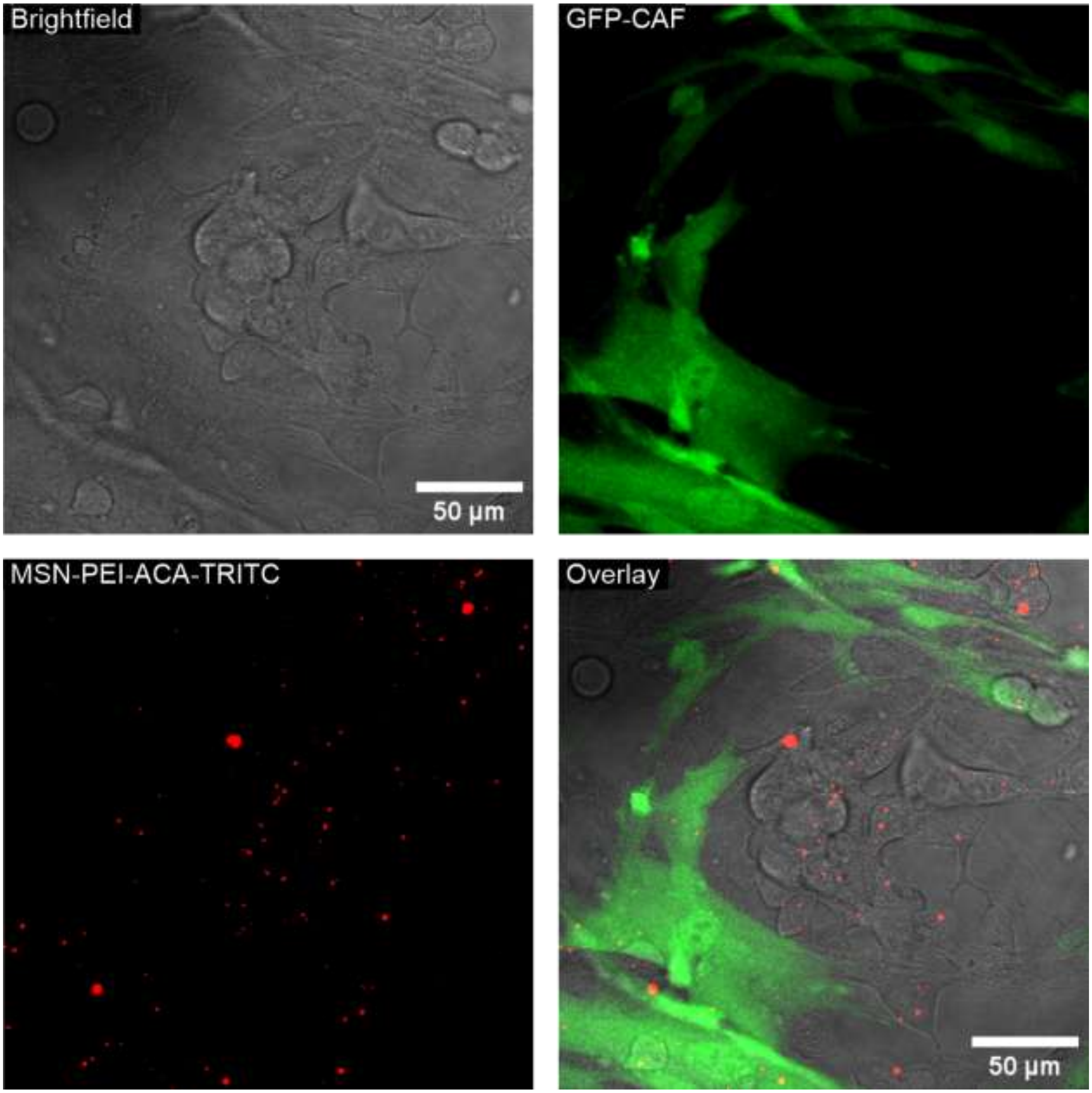

Supplementary Figure 6. Cellular uptake of ACA particles in 3D co-cultures containing LNCaP tumor cells and CAFs. MSNs were observed to nearly specifically localize with tumor cells. The GFP-tag was captured at 488 nm and TRITC at 561 nm as z-stacks with Leica confocal microscope. These channels were turned into maximum intensity projections while the brightfield channel was captured at the best focus z-position. Linear ITF modified for optimal visualization. Scale bar = 50 µm.

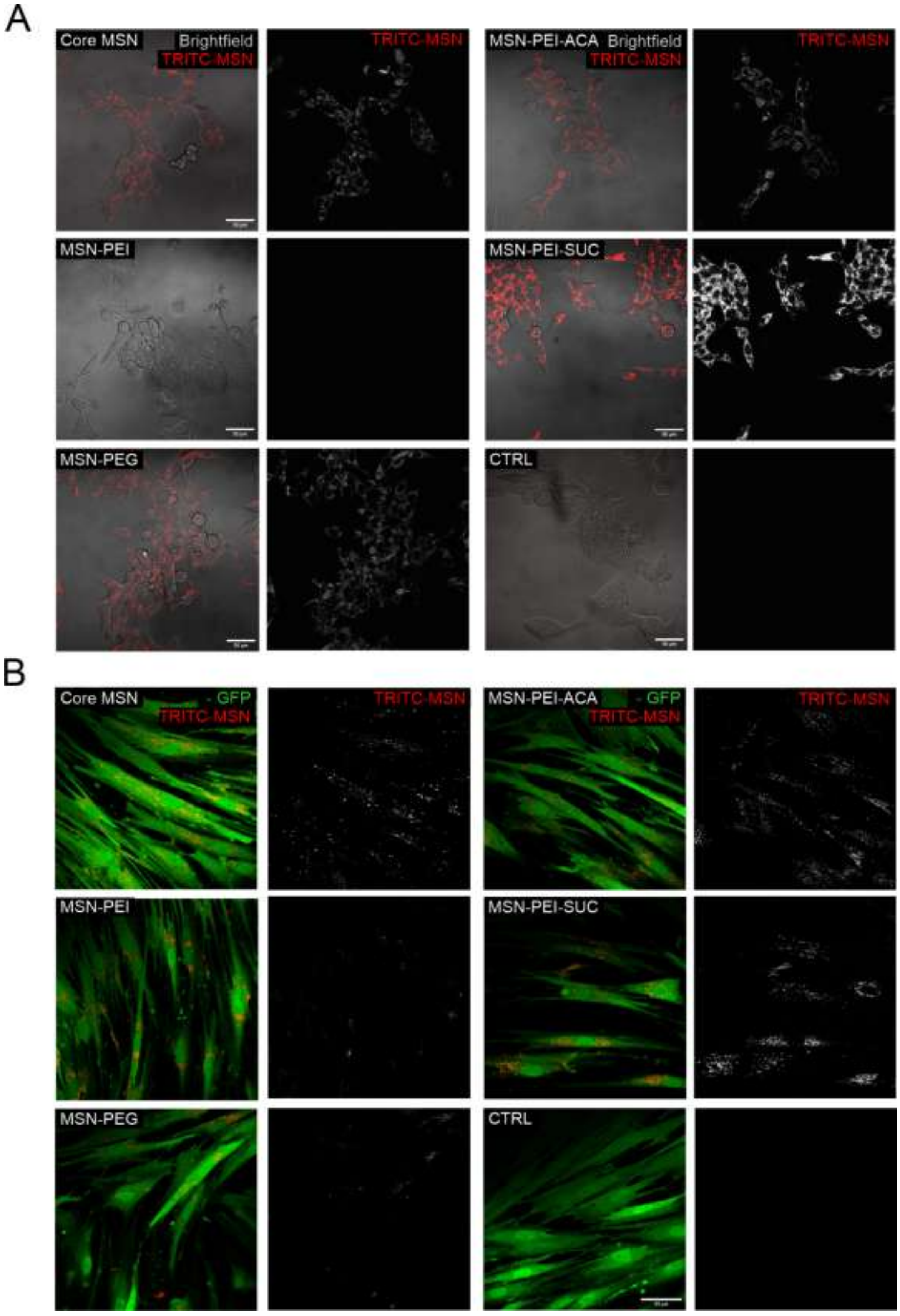
A
Core MSN
Brightfield
TRITC-MSN
TRITC-MSN
MSN-PEI-ACA
Brightfield
TRITC-MSN
TRITC-MSN
MSN-PEI
MSN-PEI-SUC
MSN-PEG
CTRL
B
Core MSN
GFP
TRITC-MSN
TRITC-MSN
MSN-PEI-ACA
GFP
TRITC-MSN
TRITC-MSN
MSN-PEI
MSN-PEI-SUC
MSN-PEG
CTRL

Supplementary Figure 7. Internalisation of TRITC-labelled MSNs into cells monocultured in collagen in 3D. Leica SP5 confocal imaging, ITF modified for visualisation, scale bar = 50 µm. Relative surface charge of MSNs are indicated with minus- and plus signs. Control is represented by untreated cells. **A)** LNCaP. Merge images consist of brightfield composited with 561 nm channel (TRITC, red, MSNs). **B)** PF179T-GFP. Merge images consist of 561 nm channel (TRITC, red, MSNs) composited with 488 nm channel (-GFP, green, CAFs). Images depicting 488 nm channel were smoothed before merging to allow optimal visualisation.

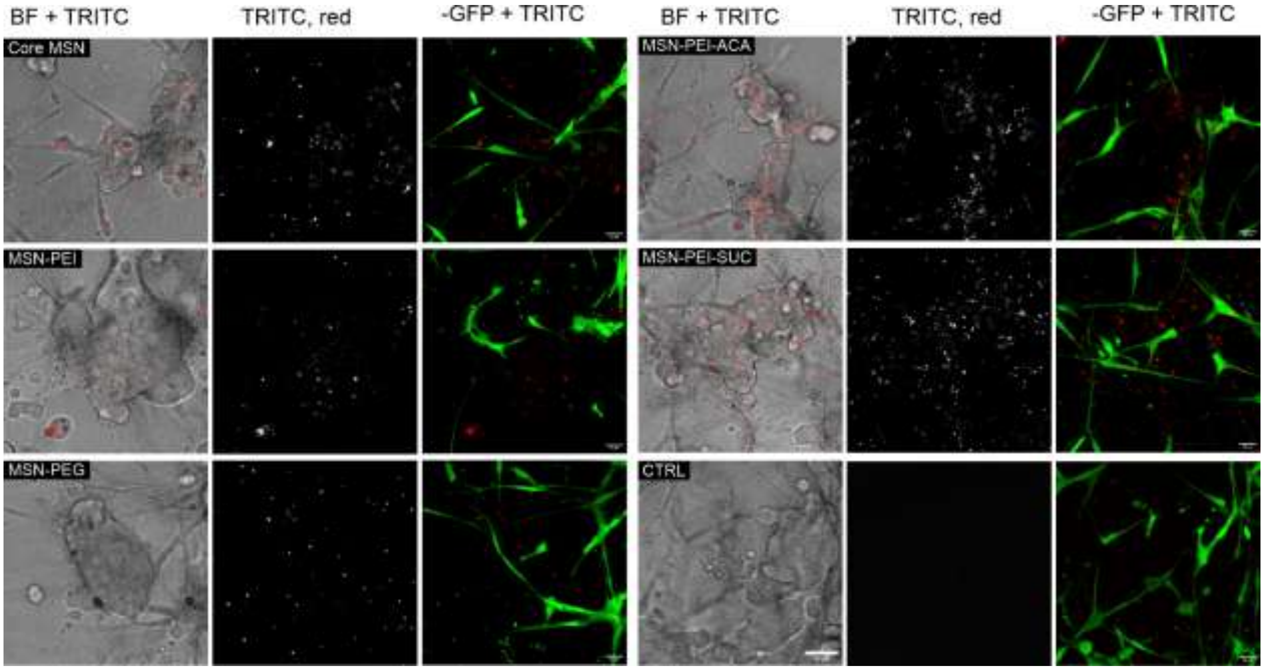

Supplementary Figure 8. MCF-7 cells (transparent) and CAFs (green) in a 3D co-culture treated with 10 µg/ml MSN derivatives. Live-cell confocal microscopy performed with 3i spinning disc. Linear ITF modified for visualization. Scale bar = 50 µm.

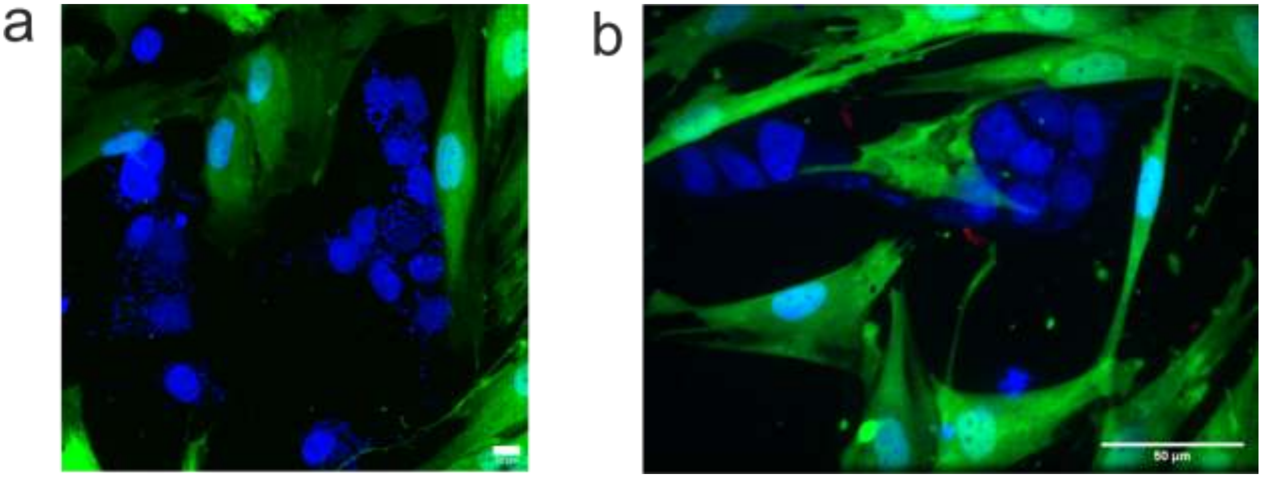

Supplementary Figure 9. Confocal images of dye release experiments where no MSNs were added, thus representing controls. GFP-CAFs captured with 488 nm laser and nuclei were dyed with Draq5, enabling capturing with 604 nm laser. 561 nm laser was used at equivalent exposure time to evaluate possible presence of autofluorescence. LNCaP and CAF co-cultures in 2D (a, scale bar = 10 µm) and 3D (b, scale bar = 50 µm).

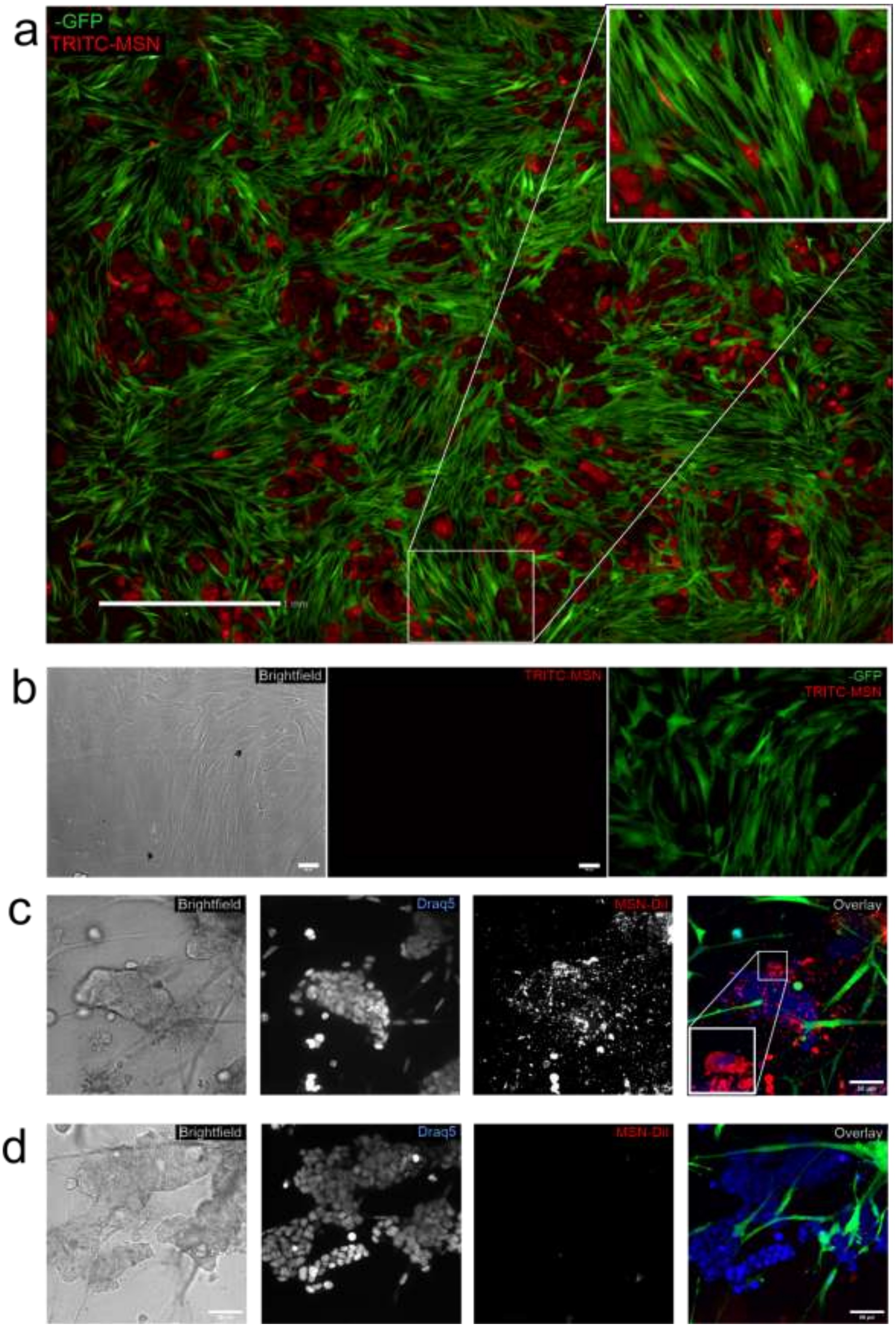
a
-GFP
TRITC-MSN
b
Brightfield
TRITC-MSN
-GFP
TRITC-MSN
c
Brightfield
Draq5
MSN-DiI
Overlay
d
Brightfield
Draq5
MSN-DiI
Overlay

Supplementary Figure 10. MSN-PEI-ACA-DiI dye release in MCF-7 co-cultures. The co-cultures were established with MCF-7 (transparent) and GFP-expressing CAFs (green). Merged images consist of far-red 604 nm (Draq5, blue LUT), 561 nm channel (DiI, red) and 488 nm channel (GFP, green). ITF modified for optimal visualization. **A)** 2D culture consisting of stitched-together images. Imaging performed with Operetta confocal automatic imaging system, scale bar = 1 mm, **(b)** represents untreated control from this experiment. **C)** 3D co-cultures of MCF-7 and CAFs in collagen, treated with 10 µg/ml MSN-PEI-ACA-DiI. **(D)** represents untreated control from this experiment. 3i confocal spinning disc imaging, scale bar = 50 µm.

A

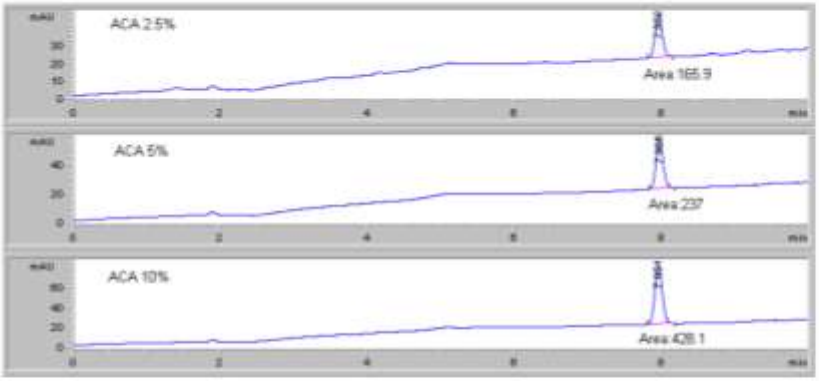

B

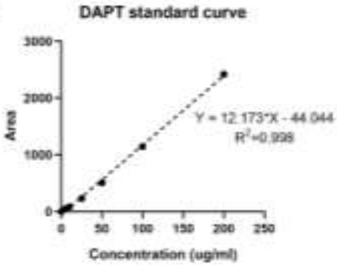

C

Theoretical loading, wt%	Loading degree measured by HPLC, wt%	Corresponding free DAPT (μM) in cell studies
2.5	1.80	0.608
5	2.38	1.216
10	3.93	2.432

Supplementary Figure 11. DAPT loading quantification by HPLC. A) Representative output signals of DAPT loaded MSN-PEI-ACA nanoparticles with different loading degrees acquired from ChemStation software. B) Calibration curve of DAPT utilized to analyze the drug loading degree. C)Theoretical and measured loading degrees of MSN-PEI-ACA.
